# Comparative bioavailability study of supplemental oral Sucrosomial**^®^** vs. oral conventional vitamin B12 in enhancing circulatory B12 levels in healthy deficient adults: a multicentre, double-blind randomized clinical trial

**DOI:** 10.3389/fnut.2024.1493593

**Published:** 2024-11-08

**Authors:** Nazia M. Memon, Gabriele Conti, Elisa Brilli, Germano Tarantino, Muhammad N. A. Chaudhry, Ameeran Baloch, Areaba Shafiq, Sami U. Mumtaz, Wafa Qaisar, Somia Iqtadar, Saida Abrar, Ayesha Kanwal, Muhammad H. Akhtar, Hina Latif, Fazle Rabbani, Ikram D. Ujjan, Silvia Turroni, Amjad Khan

**Affiliations:** ^1^Department of Pathology, Liaquat University of Medical and Health Sciences (LUMHS), Jamshoro, Pakistan; ^2^Unit of Microbiome Science and Biotechnology, Department of Pharmacy and Biotechnology, University of Bologna, Bologna, Italy; ^3^Human Microbiomics Unit, Department of Medical and Surgical Sciences, University of Bologna, Bologna, Italy; ^4^Department of R&D, PharmaNutra S.p.A, Pisa, Italy; ^5^Punjab Institute of Cardiology, Lahore, Pakistan; ^6^NIMRA Cancer Institute, Jamshoro, Pakistan; ^7^Department of Psychiatry, Lady Reading Hospital (LRH), Peshawar, Pakistan; ^8^Department of Medicine, King Edward Medical University (KEMU), Lahore, Pakistan; ^9^Gulab Devi Hospital, Lahore, Pakistan; ^10^Department of Gynecology & Obstetrics, LRH, Peshawar, Pakistan; ^11^Department of Oncology, University of Oxford, Oxford, United Kingdom

**Keywords:** Sucrosomial^**®**^ vitamin B12, Sucrosomial^**®**^ technology, vitamin B12 deficiency, oral B12 supplement, vitamin B12 bioavailability

## Abstract

**Background:**

Vitamin B12 is essential for neurological function, red blood cell formation, and DNA synthesis. Deficiency can lead to diverse health conditions, including megaloblastic anemia and neurological issues. Oral supplementation is a standard treatment for B12 deficiency. The Sucrosomial^®^ carrier system offers an innovative approach that enhances supplemental nutrient absorption and bioavailability.

**Objectives:**

This study aimed to compare the effectiveness of oral Sucrosomial^®^ vitamin B12 formulation vs various conventional B12 supplements, randomly selected from local pharmacies, in increasing and maintaining circulatory B12 levels in healthy deficient adults (200–300 pg/mL).

**Methods:**

A randomized, double-blind clinical trial was conducted across three centers in Pakistan from April to July 2024. At KEMU, participants received either Sucrosomial^®^ vitamin B12 or Mecogen SL B12; at LRH, Sucrosomial^®^ B12 or B-SUB B12; and at LUMHS, Sucrosomial^®^ B12, Evermin B12, or Neuromax B12. Participants took a daily single dose of 1,000 μg of the assigned B12 formulation for 7 days. Serum B12 levels were measured at baseline (day 0) and on days 1, 3, 5, and 7.

**Results:**

Sucrosomial^®^ B12 was significantly more effective than conventional B12 formulations in increasing and maintaining higher serum B12 levels across all time points. At KEMU, it reached a peak concentration of 454 ± 3.9 pg/mL by day 5, compared to 274 ± 11.1 pg/mL with Mecogen SL B12. At LRH, it peaked at 496 ± 34.4 pg/mL by day 5 versus 304 ± 49.4 pg/mL for B-SUB B12. At LUMHS, it reached 592.7 ± 74.3 pg/mL by day 7, compared to 407.24 ± 41.6 pg/mL for Evermin B12 and 263.82 ± 23.8 pg/mL for Neuromax B12. Sucrosomial^®^ B12 was the only formulation to surpass the deficiency-borderline threshold (200–300 pg/mL) within 24 h of the first dose and was well tolerated with no reported side effects.

**Conclusion:**

Sucrosomial^®^ vitamin B12 demonstrated superior efficacy in rapidly and consistently elevating and maintaining higher circulatory B12 levels compared to conventional supplements. Its characteristic absorption mode and proven efficacy suggest it could effectively address B12 deficiency in a broad range of populations, including those with gastrointestinal conditions and pernicious anemia, thereby supporting overall health.

**Clinical trial registration:**

clinicaltrials.gov, NCT06376591.

## 1 Introduction

**Figure 1:**
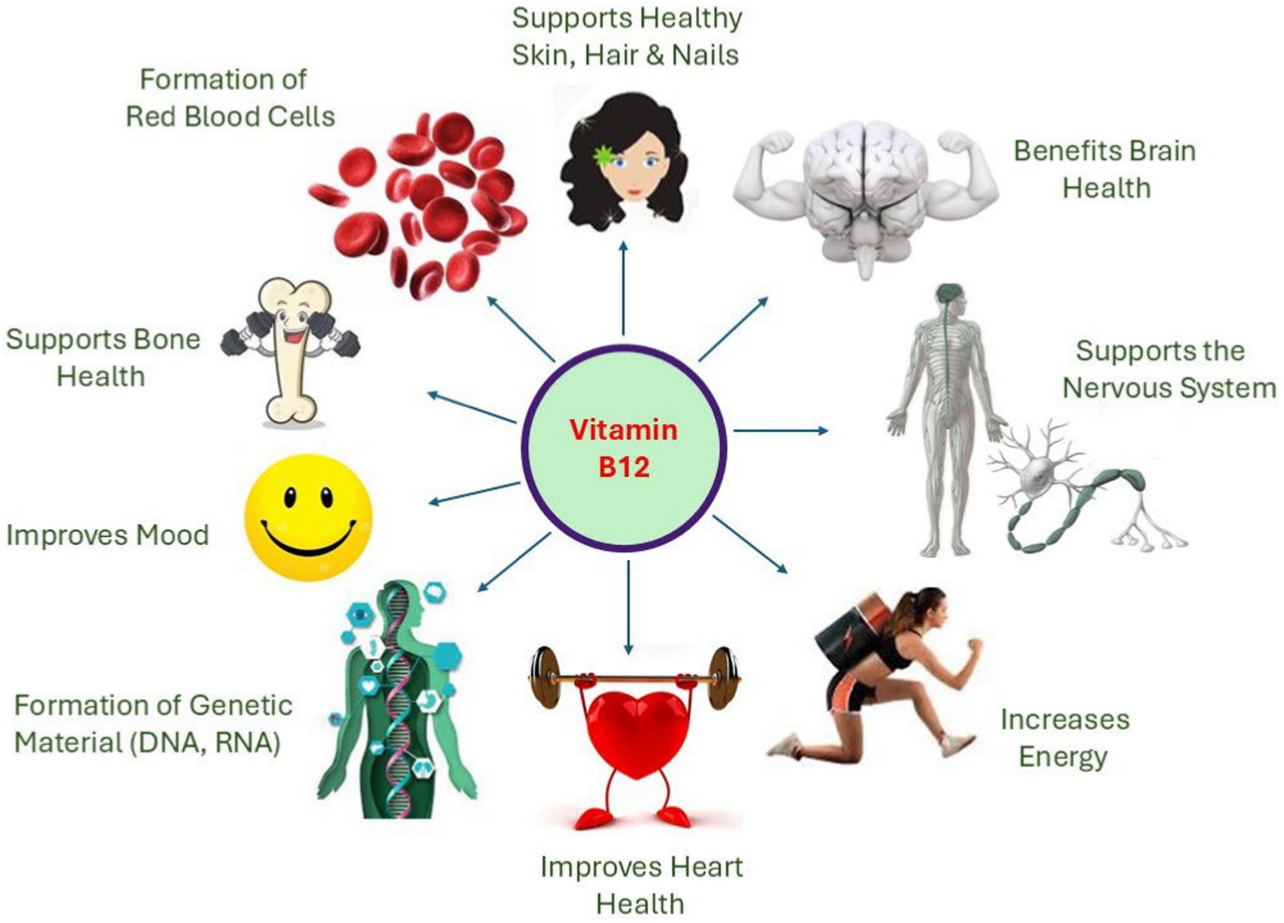
Physiological roles of vitamin B12.

Vitamin B12 (cobalamin) is a crucial water-soluble vitamin essential for various physiological processes that are vital to human health (Figure 1). It plays an indispensable role in DNA synthesis, red blood cell formation, and neurological function (1–3). Additionally, vitamin B12 is critical for cellular energy production, as it aids in the metabolism of fatty acids and amino acids (4). A key function of vitamin B12 is its role in converting homocysteine to methionine, an amino acid integral to protein synthesis, gene expression regulation, and numerous other bodily functions (5). Vitamin B12 is also essential for maintaining the integrity of the myelin sheath, which protects nerve cells and ensures efficient nerve signal transmission (6). Vitamin B12 works closely with vitamin B9 (folate or folic acid) to support red blood cell production, optimize iron utilization, and synthesize S-adenosylmethionine—a compound crucial for immune regulation and mood stabilization (7). Despite its importance, the body cannot produce sufficient vitamin B12 independently. Although certain bacteria in the human colon synthesize small amounts, the absorbed quantity is inadequate to meet the body’s needs. Consequently, obtaining bioavailable vitamin B12 from animal-derived foods such as meat, fish, poultry, eggs, and dairy products is essential for maintaining optimal health (2).

**Figure 2:**
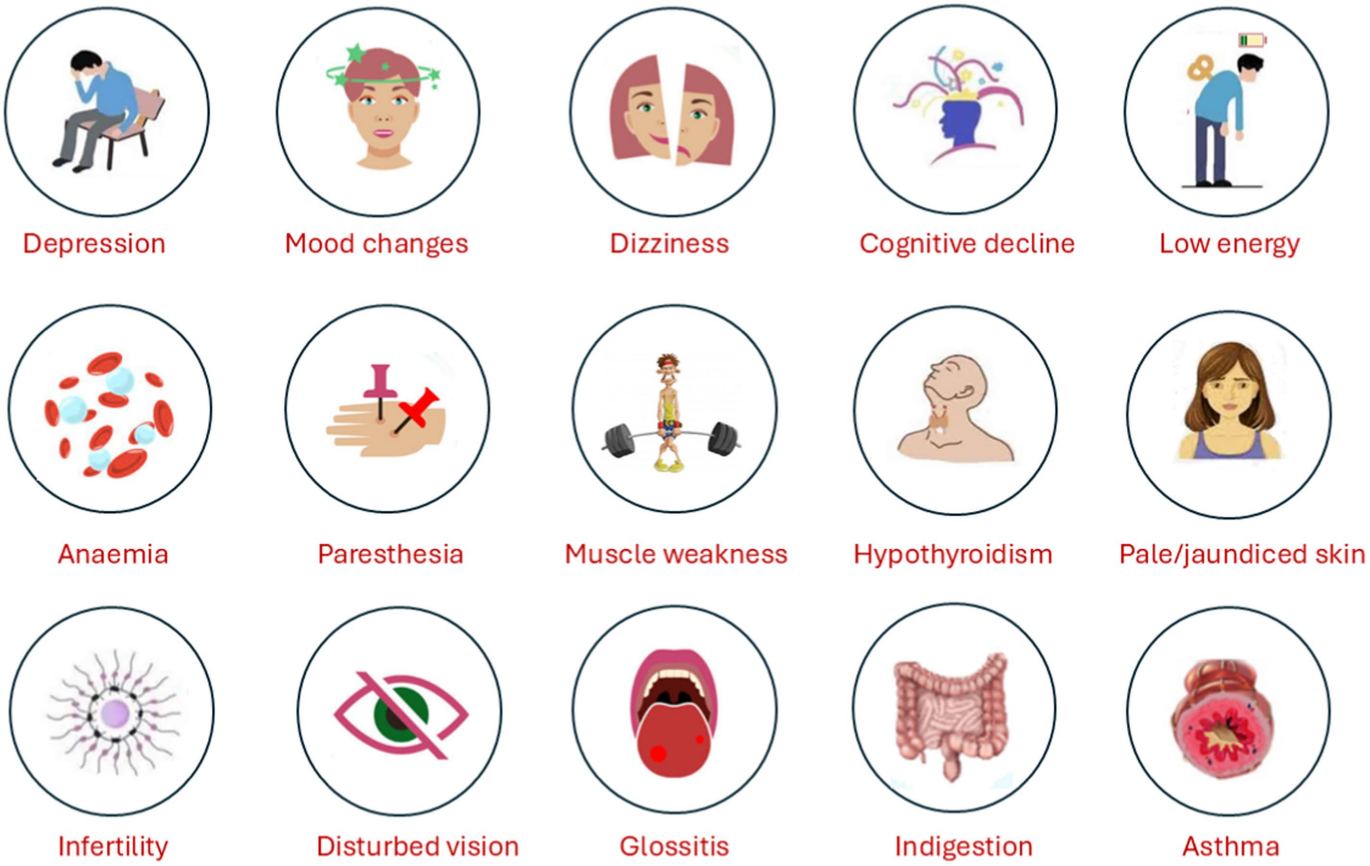
Various health conditions associated with vitamin B12 deficiency.

Vitamin B12 deficiency, defined as ciculatory B12 level below 150–200 pg/mL (borderline 200–300 pg/mL) (8), is a significant global health concern, particularly affecting the elderly (9, 10), pregnant women (11), and young children (12), especially in regions with limited dietary options. Deficiency is also common among individuals with gastrointestinal (GI) disorders and those adhering to restrictive diets like vegetarianism or veganism. This deficiency can lead to a wide array of severe health problems (Figure 2), including megaloblastic anemia and irreversible neurological damage (13). Symptoms of vitamin B12 deficiency often first manifest as hematological issues, such as pernicious anemia and megaloblastic anemia, which involve enlarged red blood cells due to impaired DNA synthesis during erythropoiesis. However, neurological symptoms and cognitive decline can occur even in the absence of anemia (14). In children, vitamin B12 deficiency can result in anemia (15–17), stunted growth (18), impaired cognitive abilities (17, 19), and developmental delays (17), potentially leading to irreversible consequences (20, 21).

For individuals at risk of B12 deficiency, treatment is often necessary to maintain adequate circulatory levels (22), with oral supplementation being a common approach. However, conventional oral B12 supplements, such as cyanocobalamin and methylcobalamin, face significant challenges during their transit through the GI tract (23). Disruptions in the intrinsic factor (IF) pathway, including impaired IF production due to autoimmune conditions (pernicious anemia), damage to the ileum from diseases like Crohn’s or celiac disease, or surgical resection of the ileum, can severely impair B12 absorption. Additionally, the harsh acidic environment of the stomach and/or dysfunctions in R-proteins (haptocorrins) that protect B12 in the stomach, and potential degradation by gastric enzymes can compromise the stability and bioavailability of oral B12 supplements. Moreover, deficiencies or dysfunctions in transcobalamin II, which is essential for transporting B12 in the bloodstream, can further impact its bioavailability. Consequently, the absorption of supplemental oral B12 can be variable and often suboptimal, particularly in individuals with compromised GI function. On the other hand, intramuscular injection of vitamin B12 is frequently used as an alternative. While this method bypasses the GI tract and generally ensures more reliable absorption, it is not without its drawbacks. Intramuscular injections can be quite painful, particularly at the injection site, and may cause mild side effects such as diarrhea, temporary itching of the skin, and a sensation of swelling. In rare cases, more severe reactions, such as anaphylaxis, may occur (24).

Thus, an optimized carrier system is crucial for enhancing the bioavailability of supplemental oral supplemental B12. A well-designed carrier system can shield B12 from degradation, facilitate its transport through the GI tract, control its release at the appropriate site in small intestine (the ileum), and promote efficient absorption. Without such a system, the absorption of supplemental B12 can be inconsistent and inadequate, particularly in individuals with compromised GI function or those with conditions that impair the production of IF.

Sucrosomial^®^ technology represents an advanced carrier system that potentially addresses these challenges. This system entraps the active agent within a lipid-based vesicle, enhancing its stability during transit through the GI tract and improving its absorption by protecting the active agent from degradation, controlling its release, and promoting its passage across the intestinal barrier. This innovative approach has been shown to enhance the absorption and subsequent bioavailability of a variety of supplemental nutrients, such as vitamin D3, iron, and magnesium, suggesting that it could similarly improve the efficacy of oral B12 supplementation (25–29).

To explore the potential advantages of Sucrosomial^®^ B12 over conventional oral B12 formulations in enhancing circulatory B12 levels, we conducted a randomized, double-blind clinical trial in healthy adults with B12 deficiency who received either Sucrosomial^®^ B12 or conventional oral B12 supplement. This study aims to provide critical insights into the effectiveness of the Sucrosomial^®^ carrier system in improving oral vitamin B12 absorption, enhancing bioavailability and, consequently, the overall efficacy of B12 supplementation.

## 2 Materials and methods

### 2.1 Study design

This study was a multi-center, prospective, double-blind, randomized clinical trial aimed to assess the absorption rate and hence bioavailibility of an innovative oral Sucrosomial^®^ B12 (Sidevit^®^ B12 developed by Pharmanutra S.p.A, Pisa, Italy) in enhancing circulatory B12 levels in healthy deficient adults, compared to various conventional oral B12 supplement formulations, selected randomly from local pharmacies at each participating center. The trial was conducted at three centers in Pakistan. At Mayo Hospital, King Edward Medical University (KEMU), Lahore (Punjab Province), efficacy of Sucrosomial^®^ B12 was compared with conventional formulated Mecogen SL B12 (Mecobalamin 1,000 μg, manufactured by Genetics Pharmaceuticals, Lahore, Pakistan). At Lady Reading Hospital (LRH), Peshawar (Khyber Pakhtunkhwa Province), efficacy of Sucrosomial^®^ B12 was compared with conventional formulated B-SUB B12 (Methylcobalamin 1,000 μg, manufactured by Bio Life^®^ Enterprises Nutraceuticals and marketed by Honing Pharmaceutical Laboratories, Rawalpindi, Pakistan). At Liaquat University of Medical & Health Sciences (LUMHS), Jamshoro (Sindh Province), efficacy of Sucrosomial^®^ B12 was compared with conventional formulated Evermin B12 (Methylcobalamin 1,000 μg, manufactured by Global Laboratories and marketed by Reliance Pharma, Islamabad, Pakistan) & Neuromax B12 (Mecobalamin 1,000 μg, manufactured by Bio Fine Pharmaceuticals, Multan, Pakistan).

The rationale for utilizing different conventional B12 supplement at each center, for comparison with Sucrosomial^®^ B12, was to ensure the reproducibility of study outcomes amidst the variability in B12 formulations. Utilizing participants from varied ethnic backgrounds across the three centers aimed to ensure a robust comparison and significantly enhance the generalizability of the study findings. This approach allowed for a comprehensive evaluation of Sucrosomial^®^ vitamin B12’s effectiveness and safety across varying formulations and demographic groups, enhancing the overall validity and applicability of the study findings.

The study was approved by the Research Ethics Committee, Liaquat University of Medical & Health Sciences, Jamshoro, Pakistan (Ref. No. LUMHS/REC/−291), and conducted in accordance with the principles of Good Clinical Practice (GCP) and the Declaration of Helsinki. Informed written consent was obtained from all participants prior to their enrollment. The study was registered in the ClinicalTrials.gov registry (Registration No. NCT06376591).

### 2.2 Study participants

Participant enrollment occurred from April 2024 to July 2024. The study population comprised healthcare professionals, including doctors, medical students, nurses, and other support staff working at the participating hospitals. Study participants had to fullfill the following specific criteria for inclusion in the study.

#### 2.2.1 Inclusion criteria

- Healthy adults, male or female, aged 18–65 years
- Stable medical conditions, including stable hepatic, renal and hematological status confirmed by the laboratory tests.
- Normal vital signs (blood pressure, heart rate, respiratory rate, temperature) and body mass index (BMI) between 18 and 30 kg/m^2^
- Suboptimal vitamin B12 status (deficient to borderline 200–300 pg/mL), defined as serum B12 levels within the lower end of the reference range (234–894 pg/mL)
- Able to provide informed written consent
- Able to comply with study procedures and follow-up visits as outlined in the protocol

#### 2.2.2 Exclusion criteria

- Known hypersensitivity or allergy to oral vitamin B12 supplements or any of its components
- Known history of cobalt allergy or sensitivity
- Severe malabsorption syndromes, including pernicious anemia or intestinal disorders affecting vitamin B12 absorption
- Current use of acetaminophen, nonsteroidal anti-inflammatory drugs, antibiotics, antacids, proton pump inhibitors (PPIs), multivitamins, or nutritional supplements containing vitamin B12
- History of gastric bypass surgery or other procedures that significantly alter GI anatomy or function
- Significant renal impairment (estimated glomerular filtration rate [eGFR] < 30 mL/min/1.73 m^2^) or hepatic impairment
- Uncontrolled or significant cardiovascular disease, including recent myocardial infarction, unstable angina, or heart failure
- History of psychiatric illness or cognitive impairment that may impair their ability to comply with study procedures or provide informed consent
- Currently enrolled in another clinical trial involving investigational products or interventions
- Pregnant or breast-feeding women
- Any other medical condition or circumstance that, in the investigator’s judgment, would compromise the safety of the participant or the integrity of the study

#### 2.2.3 Randomization and blinding

Participants were randomly assigned in a 1:1 ratio to receive either oral Sucrosomial^®^ B12 or one of the conventional oral B12 supplement specific to each center. Both participants and investigators were blinded to the group assignments to ensure data integrity and minimize bias. Randomization was conducted using a computer-generated sequence of random numbers by an independent statistician uninvolved in the study. The allocation of participants to their respective B12 groups was carried out in a concealed manner by a remote (offsite) independent centralized team.

#### 2.2.4 Vitamin B12 supplementation

Participants in both the Sucrosomial^®^ B12 and the conventional B12 supplement groups received a single dosage of 1,000 μg B12 daily for 7 days. All participants were instructed to take their oral B12 supplement at the same time each day, specifically at 10 am. Blood samples were collected by a phlebotomist of an independent diagnostic laboratory, at specified intervals to monitor circulatory (serum) B12 levels: These intervals were: day 0 (Baseline, i.e., before the first dosage), day 1 (24 h), day 3 (72 h), day 5 (120 h), and day 7 (168 h). Serum B12 levels were measured using standardized laboratory techniques. To maintain consistency, all follow-up blood samples were collected shortly before the daily B12 supplement was taken and were promptly analyzed in the diagnostic laboratory within 30 min of collection to ensure the accuracy and reliability of the serum B12 level measurements. To ensure compliance with the study protocol, participants were required to maintain a daily log of their B12 supplement intake with the study team. Additionally, participants received daily reminders from the study team via a phone call or text message to take their supplement at the designated time. Compliance was further assessed by counting the remaining tablets at each follow-up blood sample collection to verify that the participants had taken the supplement as instructed.

To assess the safety profile of the B12 supplements, participants’ liver and renal function tests, full blood count, and vital signs (including blood pressure, heart rate, respiratory rate, and body temperature) were evaluated and recorded at baseline and after the completion of 7 days of B12 supplementation.

### 2.3 Outcome measures

The primary outcome measure of the study was the bioavailibility of the B12 supplements, evaluated by the enhancement of circulatory B12 levels. Secondary outcomes encompassed the safety and tolerability of the B12 supplements, assessed through any significant negative effect on vital signs, liver function enzymes, renal function, and complete blood count, and incidence of side effects, treatment-emergent effects, and serious adverse events.

### 2.4 Statistical analysis

The Wilcoxon rank-sum test was used to compare the absorption (bioavailability) of the Sucrosomial^®^ B12 formulation with the local B12 formulations used in the study hospitals and the effect on elevating blood B12 levels. Homogeneity in gender, age, weight, and body mass index (BMI) was assessed using Pearson’s Chi-squared test with Yates’ continuity correction for dichotomous variable (gender), and either the Wilcoxon or t-test for the continuous variables (age, weight, BMI), depending on data distribution as determined by the Shapiro–Wilk test. *p*-values were adjusted for multiple comparisons using the Benjamini–Hochberg correction (False Discovery Rate - FDR) (CIT) (30). All consolidated values are presented as mean ± standard error of the mean (SEM). The Wilcoxon test or two-sample t-test was also used to assess the safety and tolerability of the vitamin B12 formulations. Statistical analyses were performed using R version 4.4.0 (CIT) (31), with the “ggplot2” package (CIT) used for graphical representations (32). Based on previous studies, a sample size of ≥20 subjects in each cohort was considered sufficient for this exploratory descriptive study (25, 33–36).

## 3 Results

The study CONSORT diagram is provided in Supplementary Figure 1. A total of 91 individuals were screened for eligibility across the three cohort studies. Of these, 15 individuals were excluded based on the study’s exclusion criteria, resulting in a final study population of 76 participants—24 in the KEMU study cohort, 22 in the LRH cohort, and 30 in the LUMHS cohort. All participants completed the study, with no dropouts reported. Participants demographic and anthropometric characteristics and baseline circulating vitamin B12 levels in the study cohorts are shown in Table 1. In the KEMU study cohort, the mean age was 38.2 ± 9.4 years, consisting of 16 males and 8 females. The LRH study had a mean age of 32.7 ± 7.4 years, with 15 females and 7 males. In the LUMHS cohort, the mean age was 35.7 ± 6.9 years, including 20 males and 10 females. In each study cohort, the groups were well balanced with respect to age, anthropometric characteristics (age, weight, BMI) and baseline circulating vitamin B12 levels (*p* > 0.05) Supplementary Table 1. Among the three cohorts, only the LUMHS cohort demonstrated a balanced gender distribution across the groups (Pearson’s Chi-square test; KEMU, *p* = 0.025; LRH, *p* = 0.004; LUMHS, *p* = 0.22).

**Table 1:** Study participants demographics, anthropometric characteristics, and baseline circulatory (serum) vitamin B12 levels.

	KEMU study		LRH study		LUMHS study		
Parameter	Sucrosomial^®^ B12 (n=12)	Mecogen SL B12 (*n*=12)	Sucrosomial^®^ B12 (*n*=10)	B-SUB B12 (*n*=12)	Sucrosomial^®^ B12 (*n*=10)	Evermin B12 (*n*=10)	Neuromax B12 (*n*=10)
Gender							
Male, *n*	9	7	2	5	6	7	7
Female, *n*	3	5	8	7	4	3	3
Age (years)	42.8 ± 2.5	33.4 ± 2.5	34.7 ± 2.4	31.2 ± 2.1	34.8 ± 2.5	36.2 ± 2.6	36.3 ± 2.6
Weight (kg)	73.6 ± 2.6	66.6 ± 4.4	68.1 ± 3.0	67.5 ± 1.9	69.6 ± 4.5	73.1 ± 4.6	70.8 ± 4.6
BMI (kg/m^2^)	25.5 ± 0.8	25.4 ± 1.3	25.6 ± 1.2	25.8 ± 0.8	24.8 ± 1.3	26.6 ± 1.8	24.6 ± 1.8
Vit. B12 (pg/ml)	191.9 ± 15.6	201.7 ± 18.4	285.1 ± 27.2	280.7 ± 44.8	225.7 ± 24.9	221.1 ± 15.4	193.1 ± 15.4

Data are presented as mean and standard error of the mean (SEM) or numbers. No statistically significant difference was observed in any variable between the Sucrosomial^®^ B12 and conventional oral vitamin B12 treatment groups within each cohort study.

### 3.1 Circulatory levels of Sucrosomial^**®**^ B12 vs. conventional Mecogen SL B12 formulation (KEMU study)

**Figure 3:**
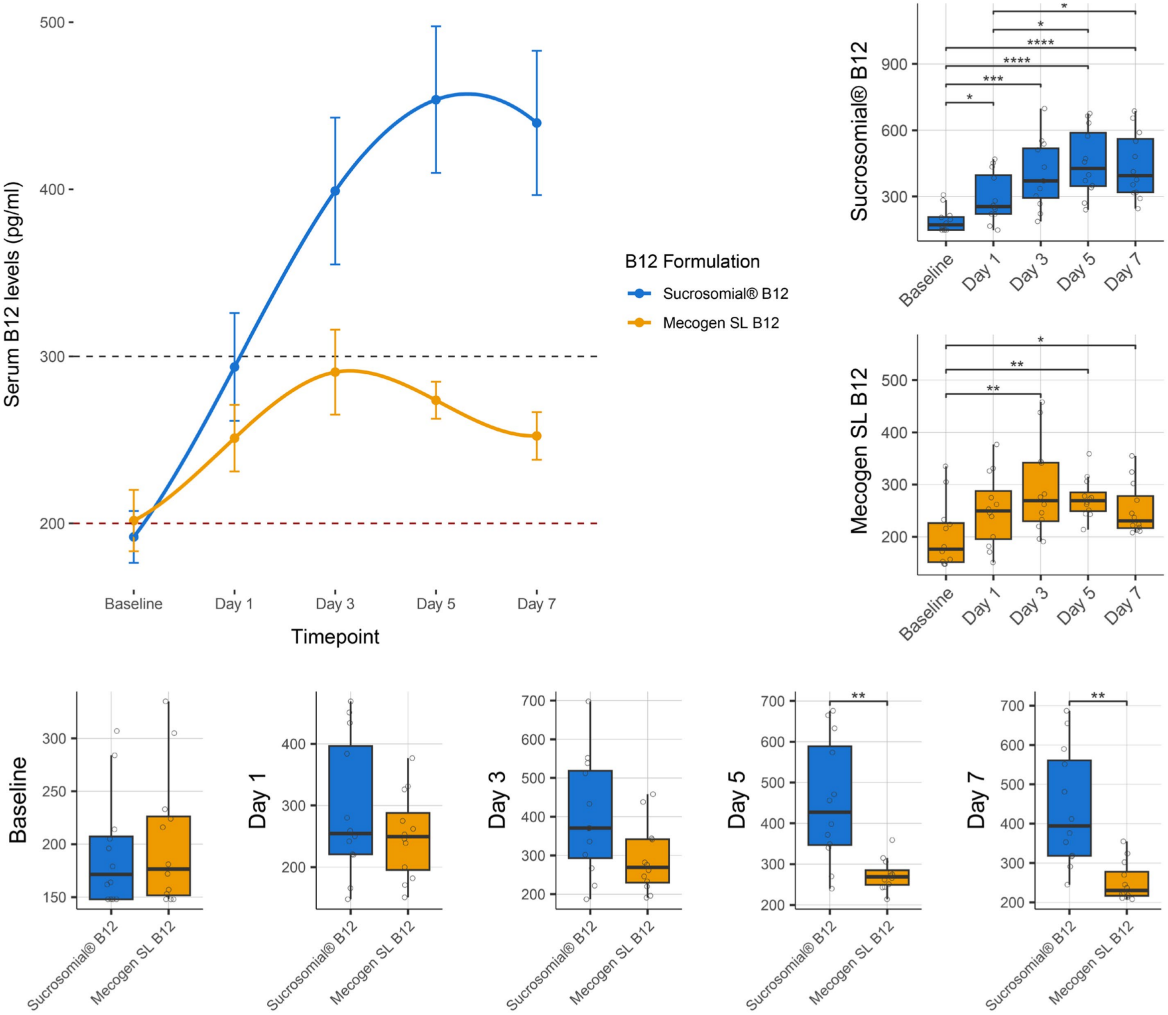
Effect of supplementation of Sucrosomial^®^ B12 or conventional oral Mecogen SL B12 formulation on circulatory (serum) vitamin B12 levels in vitamin B12-deficient (serum level < 200 pg/mL) and borderline (serum level between 200 and 300 pg/mL) healthy adults (supplement B12 dosage, one tablet 1,000 μg daily for 7 days). The top-left connected scatterplot shows participants serum B12 levels (expressed as mean ± SEM) before supplementation (baseline) and daily thereafter, with levels recorded every second day for 1 week. Sucrosomial^®^ B12, *n* = 12; Mecogen SL B12, *n* = 12. Dashed horizontal lines indicate the B12 deficiency and borderline thresholds (200 and 300 pg/mL, respectively). Boxplots show daily vitamin B12 levels recorded for each formulation, with significant differences over time (top-right) and between groups (bottom). Statistical analysis was performed using the Wilcoxon test with FDR correction; only significant differences are indicated, *p < 0.05, **p < 0.01, ***p < 0.001, ****p < 0.0001. Blood sample collection and testing were conducted by Chughtai Lab PK (https://chughtailab.com/).

Vitamin B12 levels were monitored for 1 week after treatment with either the Sucrosomial^®^ B12 formulation or the conventional Mecogen SL B12 formulation. As shown by connected scatterplot in Figure 3 (top-left), the Sucrosomial^®^ B12 formulation rapidly raised circulatory vitamin B12 levels above the deficiency threshold, exceeding 300 pg/mL (borderline level) within 24 h (day 1). Specifically, the Sucrosomial^®^ B12 formulation resulted in a significant increase in circulatory vitamin B12 levels from day 1 compared to baseline (*p* ≤ 0.012), with levels continuing to increase until day 5 (Figure 3 boxplots, top-right). In contrast, circulatory vitamin B12 levels did not exceed the borderline threshold at any time following the conventional Mecogen SL B12 formulation, despite a significant increase from day 3 to day 7 compared to baseline (*p* ≤ 0.038) (Figure 3 boxplots, top-right). Significant differences between the two formulations were observed on days 5 and 7 (*p* ≤ 0.0036), with a maximum circulatory B12 concentration of 454 ± 3.9 pg/mL measured in the Sucrosomial^®^ B12 group compared to 274 ± 11.1 pg/mL in the conventional Mecogen SL B12 group on day 5 (Figure 3 boxplots, bottom).

### 3.2 Circulatory levels of Sucrosomial^**®**^ B12 vs. conventional B-SUB B12 formulation (LRH study)

**Figure 4:**
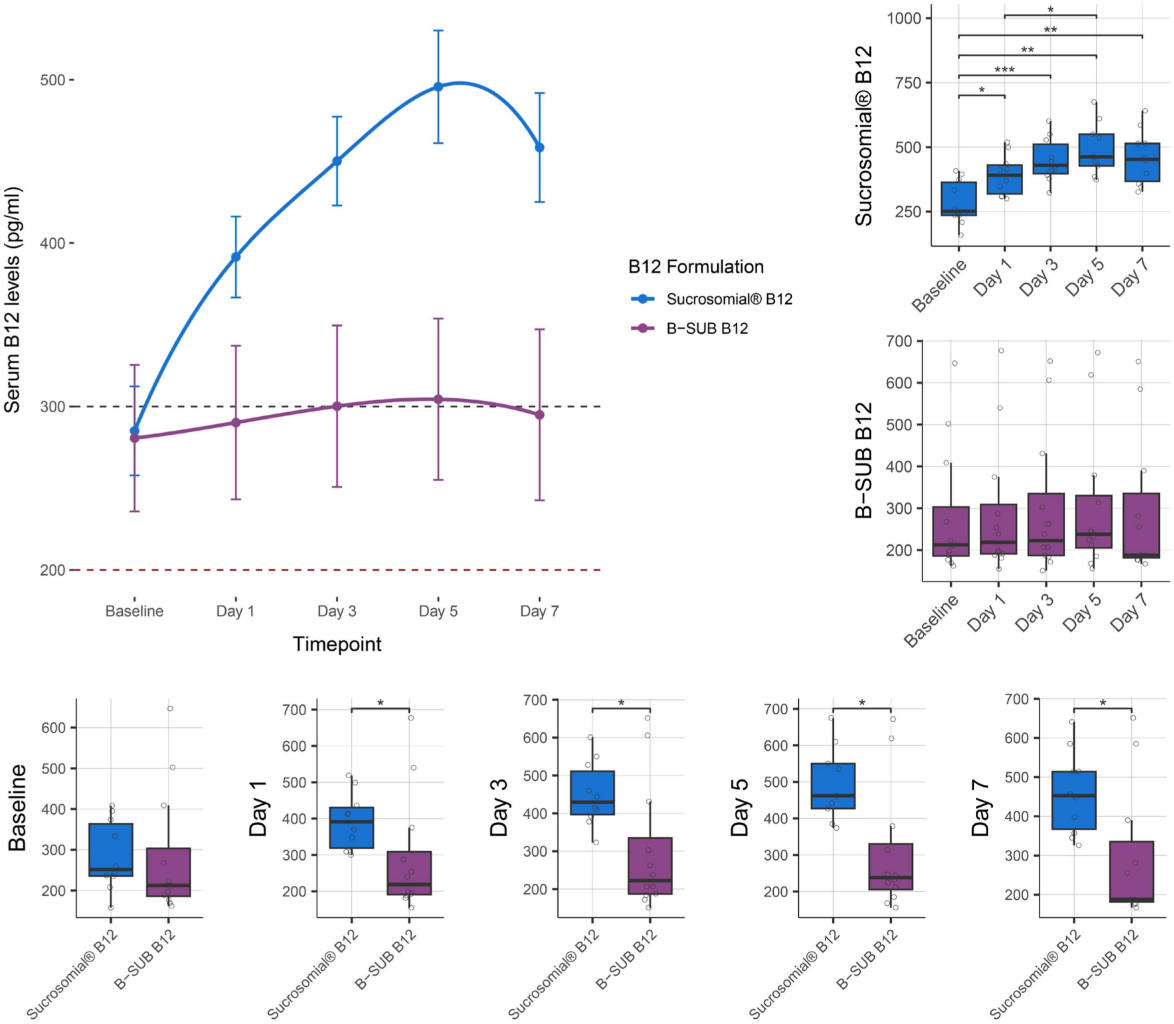
Effect of supplementation of Sucrosomial^®^ B12 vs. conventional B-SUB B12 formulation on serum vitamin B12 levels in vitamin B12-deficient (serum level < 200 pg/mL) and borderline (serum level between 200 and 300 pg/mL) healthy adults (supplement B12 dosage, one tablet 1,000 μg daily for 7 days). The top-left connected scatterplot shows participants serum vitamin B12 levels (expressed as mean ± SEM) before supplementation (baseline) and daily thereafter, with levels recorded every second day for 1 week. Sucrosomial^®^ B12, *n* = 10; B-SUB B12, *n* = 12. Dashed horizontal lines indicate the B12 deficiency and borderline thresholds (200 and 300 pg/mL, respectively). Boxplots show the daily circulatory vitamin B12 levels recorded for each formulation, with significant differences over time (top-right) and between groups (bottom). Statistical analysis was performed using the Wilcoxon test with FDR correction; only significant differences are indicated, *p < 0.05, **p < 0.01, ***p < 0.001. Blood sample collection and testing were conducted by Chughtai Lab PK (https://chughtailab.com/).

Vitamin B12 levels were monitored for 1 week after treatment with either the Sucrosomial^®^ B12 formulation or the conventional oral B-SUB B12 formulation. As shown by connected scatterplot in Figure 4 (top-left), the Sucrosomial® B12 formulation rapidly raised circulatory vitamin B12 levels above the deficiency threshold, exceeding 300 pg/mL (borderline level) from day 1. It should be noted that the baseline vitamin B12 levels in this cohort were significantly higher than in the KEMU cohort (*p* = 0.006), which may explain the faster exceeding of the vitamin B12 threshold. Similar to the KEMU study cohort, the Sucrosomial^®^ B12 formulation resulted in a significant increase in circulatory vitamin B12 levels from day 1 compared to baseline (*p* ≤ 0.015), with levels continuing to increase until day 5. In contrast, circulatory vitamin B12 levels did not exceed the borderline threshold at any time following the conventional B-SUB B12 formulation, and no significant differences were found over time (Figure 4 boxplots, top-right). In comparing the two formulations, the Sucrosomial^®^ B12 group consistently exhibited higher circulatory vitamin B12 levels at every post-treatment timepoint (*p* ≤ 0.029) (Figure 4 boxplots, bottom). On day 5, a maximum serum B12 concentration of 496 ± 34.4 pg/mL was recorded in the Sucrosomial^®^ B12 group compared to 304 ± 49.4 pg/mL in the conventional B-SUB B12 group (Figure 4 boxplots, bottom).

### 3.3 Circulatory levels of Sucrosomial^**®**^ B12 vs. conventional Evermin B12 and Neuromax B12 formulation (LUMHS study)

**Figure 5:**
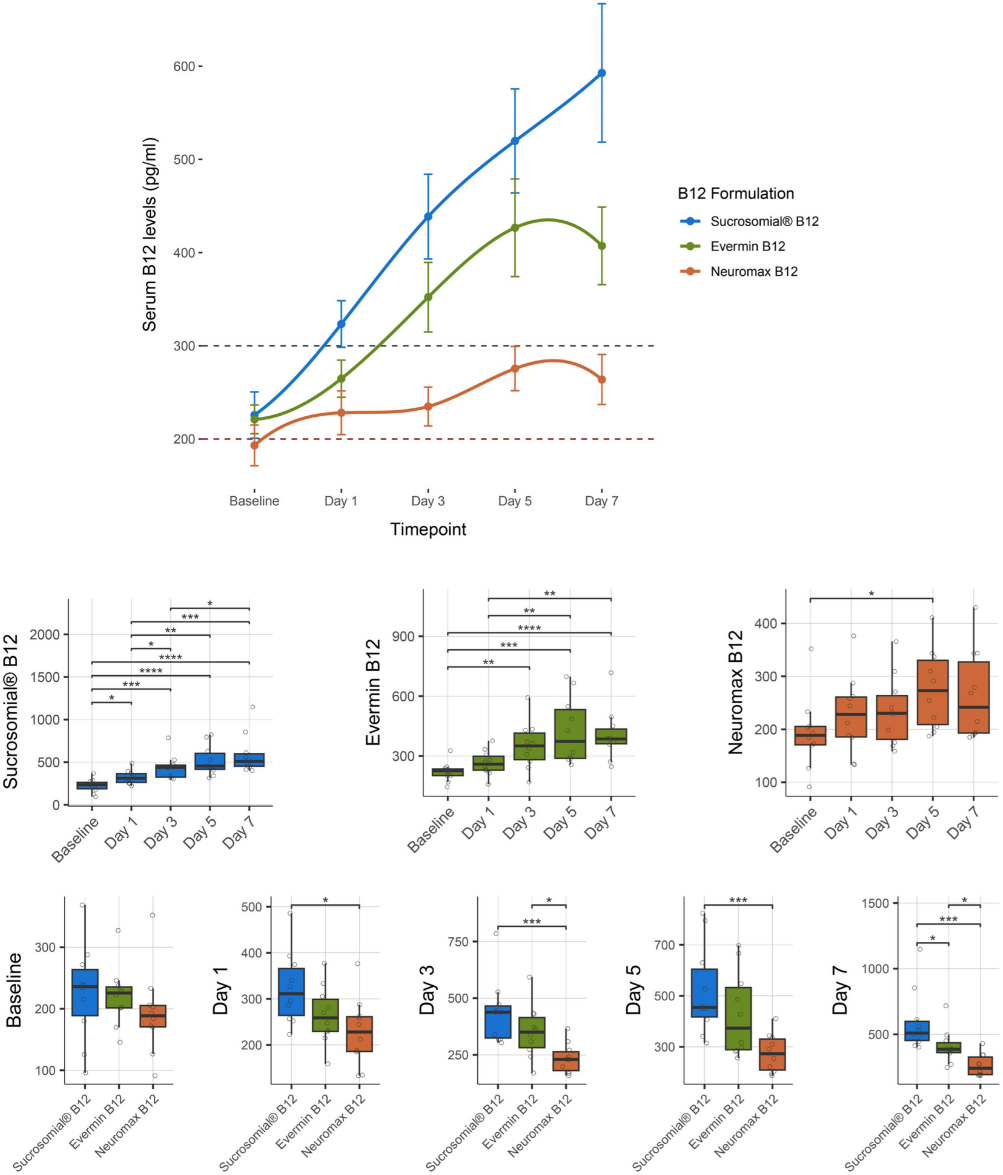
Effect of supplementation of Sucrosomial^®^ B12 vs. conventional Evermin B12 vs. Neuromax B12 formulations on serum vitamin B12 levels in vitamin B12-deficient (serum level < 200 pg/mL) and borderline (serum level between 200 and 300 pg/mL) healthy adults (supplement B12 dosage, one tablet 1,000 μg daily for 7 days). The top connected scatterplot shows circulatory vitamin B12 levels (expressed as mean ± SEM) before supplementation (baseline) and daily thereafter, with levels recorded taken every second day for 1 week. Sucrosomial^®^ B12, *n* = 10; Evermin B12, *n* = 10, Neuromax B12, *n* = 10. Dashed horizontal lines indicate the B12 deficiency and borderline thresholds (200 and 300 pg/mL, respectively). Boxplots show daily vitamin B12 levels for each B12 formulation, with significant differences over time (centre) and between groups (bottom). Statistical analysis was performed using the Wilcoxon test with FDR correction; only significant differences are indicated, *p < 0.05, **p < 0.01, ***p < 0.001. Blood sample collection and testing were conducted by Diagnostic and Research Laboratory, LUMHS, PK (https://drlab.lumhs.edu.pk/).

Vitamin B12 levels were monitored for 1 week after treatment with the Sucrosomial^®^ B12 formulation or the conventional formulations Evermin B12 and Neuromax B12. As shown in the connected scatterplot in Figure 5 (top), the Sucrosomial^®^ B12 formulation rapidly raised circulatory vitamin B12 levels above the deficiency threshold, exceeding 300 pg/mL (borderline level) from day 1, and demonstrated a similar pattern of increasing circulatory vitamin B12 levels over time as observed in the KEMU and LRH studies cohorts, reaching a peak value of 592.7 ± 74.3 at day 7, with all measurements throughout the week being significantly higher than baseline (*p* ≤ 0.012). The conventional Evermin B12 formulation showed a similar trend, with a significant overall increase in circulatory vitamin B12 levels from day 3 reaching to a peak value of 407.2 ± 41.6 at day 7 compared to baseline (*p* ≤ 0.004). Of the three formulations, the conventional Neuromax B12 was the least effective in raising circulatory vitamin B12 levels, with a significant increase only on day 5 (275.5 ± 23.8 mg/mL) (*p* = 0.018) and never exceeding the borderline threshold (Figure 5 boxplots, center). Compared to Evermin B12, the Sucrosomial^®^ B12 formulation showed a significant increase in circulatory vitamin B12 levels only on day 7 (*p* = 0.045), suggesting a similar effect in the short term, but a more sustained effect over time for Sucrosomial^®^ B12. Compared to Neuromax B12, Sucrosomial^®^ B12 resulted in significantly higher circulatory vitamin B12 levels at every post-treatment timepoint (*p* ≤ 0.023). Notably, Evermin B12 showed a generally better effect in raising vitamin B12 levels compared to Neuromax B12, with significant differences from day 3 (*p* ≤ 0.029) (Figure 5 boxplots, bottom).

### 3.4 Safety and tolerability of the B12 supplements

All the B12 supplements were generally well tolerated by all participants in the three studies. No serious adverse events, side effects, or treatment-emergent effects were reported. Furthermore, there were no significant negative impacts on liver function, renal function, hematology, or vital signs—during or after the supplementation period Supplementary Tables 2–4. The only exception was the conventional Neuromax B12 formulation, which led to significant changes in certain renal function parameters, such as serum levels of sodium, potassium, chloride, and bicarbonate, as well as in some hematological parameters, including the relative abundance of monocytes and basophils.

## 4 Discussion

In this multicenter, randomized, double-blind clinical trial, we assessed the efficacy and bioavailability of an innovative oral Sucrosomial^®^ B12 supplement, administered as a single daily dose of 1,000 μg over a 7-day period. The study compared Sucrosomial^®^ B12 with various conventional oral B12 supplements (Mecogen SL B12, B-SUB B12, Evermin B12, Neuromax B12, randomly selected from local pharmacies) across three cohorts of healthy adults with deficient to borderline B12 levels. The primary outcome was the change in serum B12 levels, following supplemental B12 intake, allowing for a comparative evaluation of the different formulations’ effectiveness in increasing and maintaining B12 concentrations in the bloodstream. Our findings reveal that Sucrosomial^®^ B12 was significantly more effective at increasing and maintaining higher circulatory B12 levels than all the four conventional B12 formulations, across all time points (day 1, day 3, day 5, and day 7). In the KEMU study cohort, Sucrosomial^®^ B12 achieved a peak circulatry B12 concentration of 454 ± 3.9 pg/mL by day 5, compared to 274 ± 11.1 pg/mL with conventional Mecogen SL B12. In the LRH cohort, it reached a peak concentration of 496 ± 34.4 pg/mL by day 5, versus 304 ± 49.4 pg/mL for B-SUB B12. In the LUMHS cohort, Sucrosomial^®^ B12 also demonstrated superior effectiveness, achieving a peak value of 592.7 ± 74.3 pg/mL on day 7, compared to 407.2 ± 41.6 pg/mL for Evermin B12 and 263.8 ± 23.8 pg/mL for Neuromax B12. Notably, Sucrosomial^®^ B12 was the only formulation to rapidly increase circulatory B12 levels from day 1 (whithin 24 h), consistently exceeding the deficiency-borderline threshold (200–300 pg/mL), whereas conventional formulations did not achieve similar efficacy. Furthermore, Sucrosomial^®^ B12 was well-tolerated, with no reported side effects, serious adverse events, or treatment-emergent effects, no negative effect on the vital signs, confirming its excellent safety and tolerability. These results underscore the superior efficacy of Sucrosomial^®^ B12 in rapidly and consistently elevating and maintaining higher circulatory vitamin B12 levels compared to conventional B12 formulations.

**Figure 6:**
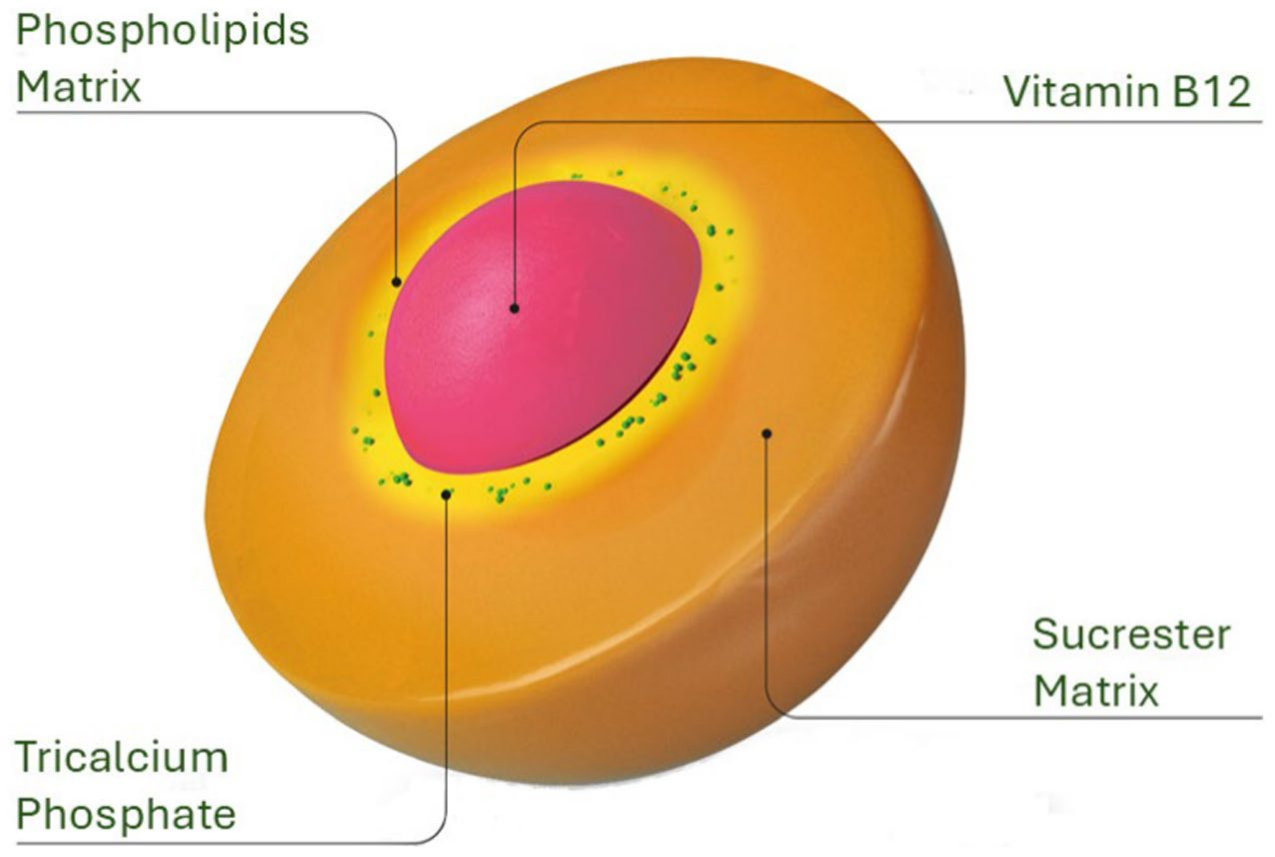
Structural design of the Sucrosomial^®^ delivery system. The innovative Sucrosomial^®^ B12 formulation, depicted here, was evaluated in the present study for its efficacy in enhancing the bioavailability of supplemental oral B12 compared to conventional oral B12 formulations.

Conventional oral B12 formulations often face significant potential challenges, including its primary reliance on IF for absorption in the Ileum, and potential degradation by stomach harsh acidic envionment, and gastric enzymes. The absorption process is significantly hindered in conditions such as pernicious anemia, Crohn’s disease, celiac disease, and following ileal surgical resection, all of which can impair B12 absorption and contribute to suboptimal supplementation outcomes. The Sucrosomial^®^ B12 formulation possibly overcomes these obstacles by entrapping vitamin B12 within a protective matrix composed of phospholipids, primarily derived from sunflower lecithin, and a sucrester (sucrose ester). This matrix, combined with additional stabilizing ingredients like starch and tricalcium phosphate, forms a robust “sucrosome” carrier system (Figure 6). The gastro-resistant properties of the sucrosome shields the vitamin from premature release, degradation and minimizing interactions with food (27), as it passes through the stomach, ensuring that the B12 reaches the intestinal mucosa intact. Once in the ileum, Sucrosomial^®^ B12 possibly bypass the traditional IF-dependent absorption pathway. The intact sucrosome vesicles facilitate absorption through both paracellular and transcellular routes, potentially involving uptake by enterocytes and M cells (27–29, 37). The formulation’s unique structure allows for passive diffusion across the intestinal epithelium, which can occur in both the jejunum and ileum. After being absorbed, B12 is gradually released from the sucrosome vesicles within the enterocytes and then transported into the bloodstream, leading to elevated circulatory B12 levels.This mechanism ensures that Sucrosomial^®^ B12 is absorbed efficiently, even in the presence of compromised IF function or damage to the ileum. As a result, this approach improves the bioavailability of oral supplemental B12, providing a more reliable source of the nutrient. However, it is worth noting that there is a possibility that some Sucrosomial^®^ B12 may enter the bloodstream in its intact form and potentially be released at the hepatocytes, this aspect of its absorption and metabolism requires further investigation.

The clinical relevance of Sucrosomial^®^ B12 is substantial, offering an innovative and effective solution for managing vitamin B12 deficiency across various populations. This advanced formulation is particularly beneficial for individuals at increased risk of deficiency, including the elderly, vegetarians, vegans, and those with GI conditions that hinder B12 absorption, such as pernicious anemia, celiac disease, Crohn’s disease, and irritable bowel syndrome (IBS), among others. The Sucrosomial^®^ carrier system significantly enhances bioavailability, allowing B12 to be absorbed more efficiently, possibly bypassing the IF pathway. This makes the Sucrosomial^®^ carrier system a compelling option for maintaining optimal B12 levels, thereby supporting overall health and preventing a wide range of health conditions linked to B12 deficiency (Figure 2). These conditions include megaloblastic anemia, neurological disorders, cognitive decline, cardiovascular issues, and complications during pregnancy (Figure 2), among others. By providing a more reliable and effective means of supplementation, Sucrosomial^®^ B12 could play a crucial role in improving patient outcomes and enhancing quality of life.

The Sucrosomial^®^ carrier system has been previously investigated for its potential to enhance the bioavailability of various nutrients, including iron, magnesium, and vitamin D3, providing a basis for its application in vitamin B12 supplementation. For instance, in a study involving vitamin D-deficient adults, the use of Sucrosomial^®^ vitamin D3 resulted in significantly higher and more efficient increases in circulatory vitamin D levels compared to conventional oral supplements (25). Similarly, iron supplementation using this system has demonstrated high bioavailability and low GI toxicity, particularly in patients with conditions like chronic kidney disease, inflammatory bowel disease, celiac disease, and those undergoing bariatric surgery (27–29). One study also found it to be as effective as intravenous iron in treating anemia in patients with ulcerative colitis (38). Additionally, research on magnesium supplementation indicated that this delivery method achieved significantly higher blood magnesium levels compared to conventional oral supplements (26). These findings suggest that this carrier system could be beneficial in enhancing the bioavailability of vitamin B12 and addressing nutrient deficiencies across various health conditions.

Our study is not free from limitations, which can be addressed in future studies. Specifically, the relatively small sample size, the unbalanced male/female ratio (with a higher number of males in two of the three cohorts), and the short intervention period constrained our ability to assess the long-term effects of the Sucrosomial^®^ B12 formulation. It has been suggested that males may be more susceptible to vitamin B12 deficiency (39), which is relevant given that we found lower baseline vitamin B12 levels in the two groups of subjects in the LUMHS cohort who received conventional B12 formulations, both of which included more males. While this may have partially biased our results, the baseline difference was not statistically significant, further strengthening the evidence for the efficacy of Sucrosomial^®^ B12. Additionally, hormonal changes associated with menopause and menstruation may influence vitamin B12 absorption and metabolism, potentially contributing to vitamin B12 deficiency. The inclusion of participants experiencing these hormonal changes may be another limitation of the study.

Despite the above limitations, the study’s strengths are notable. In a randomized, dounble-blind clinical trial, we have shown that the innovative Sucrosomial^®^ B12 formulation rapidly achieve considerably higher and maintained circulatory B12 levels, overcoming the deficiency-borderline threshold (200–300 pg/mL) within 24 h with a single 1,000 μg dose. This was observed in diverse population cohorts with multiple ethnic backgrounds, enhancing the generalizability of the results. Future research should focus on exploring the efficacy of the Sucrosomial^®^ B12 formulation in individuals with IF deficiency, such as those with pernicious anemia and GI conditions, such as Crohn’s disease or celiac disease. Additionally, investigating the formulation’s effectiveness in pediatric populations and individuals with neurological conditions could provide further insights into its benefits and safety profile.

## 5 Conclusion

In conclusion, the Sucrosomial^®^ B12 formulation represents a significant advancement in oral vitamin B12 supplementation. This innovative delivery system rapidly increases circulatory B12 levels and achieves optimal circulatory vitamin B12 status without causing any GI or other side effects. The formulation offers a reliable and efficient solution for maintaining adequate vitamin B12 intake, and supporting health in diverse populations at risk of deficiency. Its unique benefits underscore its role as an effective therapeutic strategy for managing vitamin B12 deficiency.

## Data Availability

The original contributions presented in the study are included in the article/Supplementary material, further inquiries can be directed to the corresponding author.
