# Supplementary material for: Comparative bioavailability study of supplemental oral Sucrosomial® vs. oral conventional vitamin B12 in enhancing circulatory B12 levels in healthy deficient adults: a multicentre, double-blind randomized clinical trial
Source: Front Nutr. 2024 Nov 8;11:1493593. doi: 10.3389/fnut.2024.1493593 (PMC11581850; doi:10.3389/fnut.2024.1493593)
Supplement: Supplementary file 1 [file Data_Sheet_1.pdf]

Supplementary information

Figure S1. Study CONSORT Flow Diagram

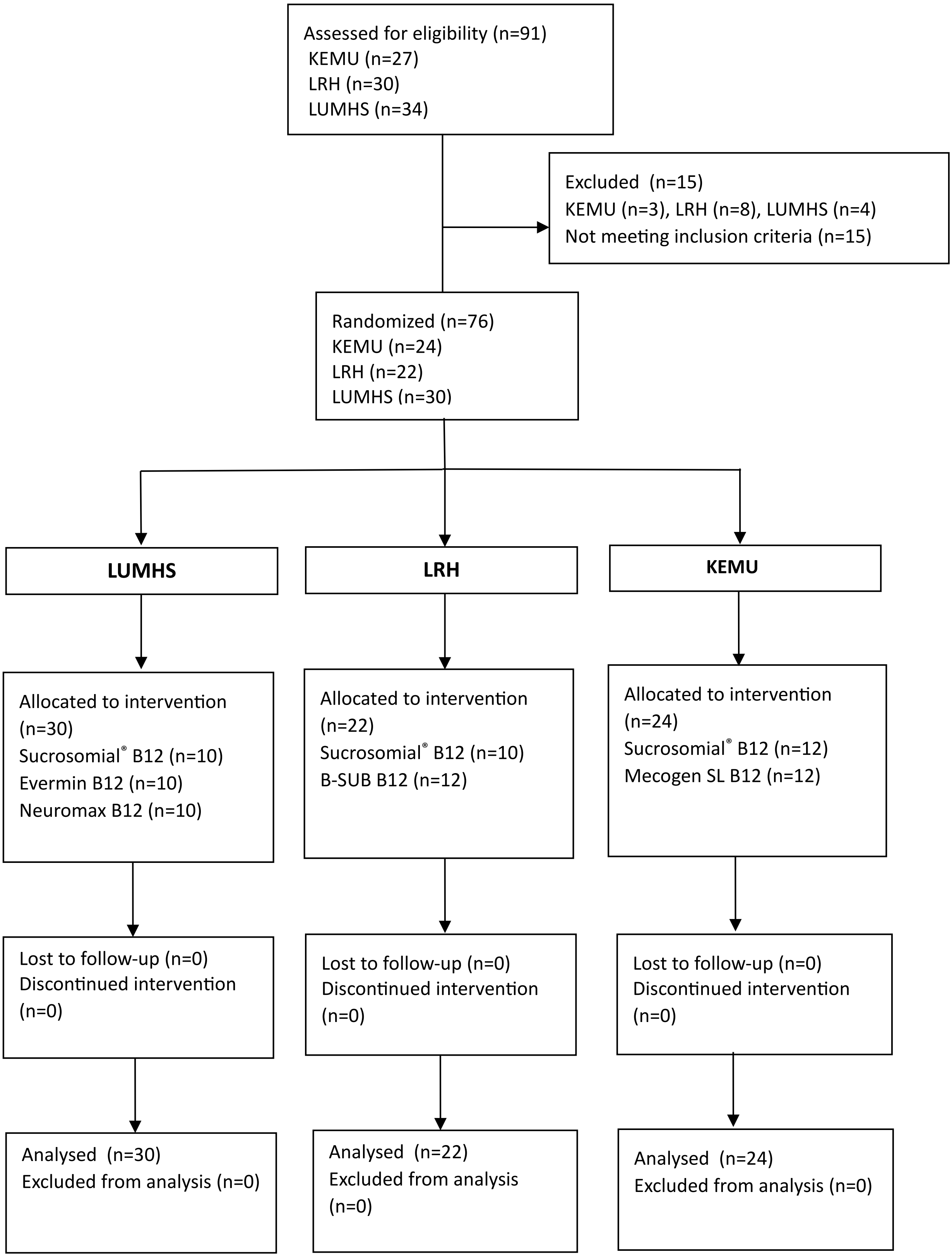

Table S1. Participants demographics, anthropometric characteristics and baseline circulating (serum) vitamin B12 levels.

KEMU cohort	Sucrosomial® B12 (n=12; 3 F, 9 M)	Mecogen SL B12 (n=12; 5 F, 7 M)	shapiro p.val	wilcoxon p.val	t. student p.val	considered p.val	fdr	sign
**Parameter**	**mean ± SEM**	**mean ± SEM**						
Age	42.8 ± 2.5	33.4 ± 2.5	9.48E-01	2.63E-02	1.35E-02	1.35E-02	6.75E-02	
Weight (kg)	73.6 ± 2.6	66.6 ± 4.4	5.63E-01	2.48E-01	1.84E-01	1.84E-01	3.07E-01	
BMI (kg/m^2)	25.5 ± 0.8	25.4 ± 1.3	5.81E-01	7.99E-01	9.62E-01	9.62E-01	9.62E-01	
Vit. B12 (pg/ml)	191.9 ± 15.6	201.7 ± 18.4	4.82E-04	6.61E-01	6.89E-01	6.61E-01	8.26E-01	

LRH cohort	Sucrosomial® B12 (n=10; 8 F, 2 M)	B-SUB B12 (n=12; 7 F, 5 M)	shapiro p.val	wilcoxon p.val	t. student p.val	considered p.val	fdr	sign
**Parameter**	**mean ± SEM**	**mean ± SEM**						
Age	34.7 ± 2.4	31.2 ± 2.1	1.89E-01	3.38E-01	2.84E-01	2.84E-01	8.88E-01	
Weight (kg)	68.1 ± 3.0	67.5 ± 1.9	8.23E-02	9.47E-01	8.70E-01	8.70E-01	8.88E-01	
BMI (kg/m^2)	25.6 ± 1.2	25.8 ± 0.8	7.42E-01	1.00E+00	8.88E-01	8.88E-01	8.88E-01	
Vit. B12 (pg/ml)	285.1 ± 27.2	280.7 ± 44.8	2.14E-03	4.18E-01	9.34E-01	4.18E-01	8.88E-01	

LUMHS cohort	Sucrosomial® B12 (n=10; 4 F, 6 M)	Evermin B12 (n=10; 3 F, 7 M)	shapiro p.val	wilcoxon p.val	t. student p.val	considered p.val	fdr	sign
**Parameter**	**mean ± SEM**	**mean ± SEM**						
Age	34.8 ± 2.5	36.2 ± 2.6	5.02E-01	9.09E-01	6.99E-01	6.99E-01	8.74E-01	
Weight (kg)	69.6 ± 4.5	73.1 ± 4.6	5.20E-01	5.70E-01	5.93E-01	5.93E-01	8.74E-01	
BMI (kg/m^2)	24.8 ± 1.3	26.6 ± 1.8	6.63E-01	3.45E-01	4.47E-01	4.47E-01	8.74E-01	
Vit. B12 (pg/ml)	225.7 ± 24.9	221.1 ± 15.4	6.92E-01	6.31E-01	8.77E-01	8.77E-01	8.77E-01	

LUMHS cohort	Sucrosomial® B12 (n=10; 4 F, 6 M)	Neuromax B12 (n=10; 3 F, 7 M)	shapiro p.val	wilcoxon p.val	t. student p.val	considered p.val	fdr	sign
**Parameter**	**mean ± SEM**	**mean ± SEM**						
Age	34.8 ± 2.5	36.3 ± 1.7	3.15E-01	8.19E-01	6.23E-01	6.23E-01	9.20E-01	
Weight (kg)	69.6 ± 4.5	70.8 ± 3.3	7.33E-02	9.40E-01	8.31E-01	8.31E-01	9.20E-01	
BMI (kg/m^2)	24.8 ± 1.3	24.6 ± 1.1	6.22E-01	9.70E-01	9.20E-01	9.20E-01	9.20E-01	
Vit. B12 (pg/ml)	225.7 ± 24.9	193.1 ± 21.8	5.07E-01	1.65E-01	3.39E-01	3.39E-01	9.20E-01	

LUMHS cohort	Neuromax B12 (n=10; 3 F, 7 M)	Evermin B12 (n=10; 3 F, 7 M)	shapiro p.val	wilcoxon p.val	t. student p.val	considered p.val	fdr	sign
**Parameter**	**mean ± SEM**	**mean ± SEM**						
Age	36.3 ± 1.7	36.2 ± 2.6	1.63E-02	8.49E-01	9.74E-01	8.49E-01	8.49E-01	
Weight (kg)	70.8 ± 3.3	73.1 ± 4.6	9.30E-01	7.62E-01	6.91E-01	6.91E-01	8.49E-01	
BMI (kg/m^2)	24.6 ± 1.1	26.6 ± 1.8	7.45E-01	3.53E-01	3.82E-01	3.82E-01	8.49E-01	
Vit. B12 (pg/ml)	193.1 ± 21.8	221.1 ± 15.4	2.14E-01	1.99E-01	3.12E-01	3.12E-01	8.49E-01	

Data are presented as mean and standard error of the mean (SEM). Both Wilcoxon and t-tests were performed. In the 'Considered p.val' column, either the Wilcoxon or t-test p-value is provided, based on the result of the Shapiro–Wilk test for data distribution (if Shapiro $p < 0.05$, the Wilcoxon p-value is reported; otherwise, the t-test p-value is selected). The considered p-values were then adjusted using FDR (false discovery rate using the Benjamini–Hochberg method), and the corresponding significance levels are indicated in the 'sign' column. BMI, body mass index; F, female; M, male.

Table S2. Safety assessement of Sucrosomial® B12 on partcipant's haematology and vital signs in the KEMU cohort (n=12).

Sucrosomial® B12 (KEMU cohort)	Baseline		Day 7		shapiro	wilcoxon	t. student	considered	fdr	Sign.
Parameters	mean	SEM	mean	SEM	p.val	p.val	p.val	p.val		
**Haematology**										
Hb (R.R 11.5-16 g/dl)	13.9	0.6	13.9	0.5	1.75E-01	9.08E-01	9.83E-01	9.83E-01	9.83E-01	
Total RBCs (R.R 4-6 × 10^12/l)	4.8	0.2	4.9	0.2	1.85E-01	5.82E-01	6.51E-01	6.51E-01	9.82E-01	
HCT (R.R 36-46%)	41.0	1.5	40.8	1.4	1.37E-01	9.08E-01	9.34E-01	9.34E-01	9.83E-01	
MCV (R.R 75-95 fl)	85.8	2.5	83.3	2.6	2.30E-03	3.39E-01	5.11E-01	3.39E-01	9.82E-01	
MCH (R.R 26-32 pg)	29.1	0.8	28.4	0.9	1.75E-04	4.93E-01	5.84E-01	4.93E-01	9.82E-01	
MCHC (R.R 30-35 g/dl)	33.8	0.2	33.9	0.3	3.20E-03	7.07E-01	8.05E-01	7.07E-01	9.82E-01	
RDW CV (R.R 11.5-14 5%)	15.0	0.4	14.6	0.6	4.34E-04	1.26E-01	5.80E-01	1.26E-01	9.30E-01	
Platelet count (R.R 150-400 × 10^9/l)	218.0	8.0	261.5	8.2	9.50E-01	2.67E-03	9.57E-04	9.57E-04	2.39E-02	*
MPV (R.R 7-11 fl)	11.1	0.3	10.9	0.3	6.51E-01	8.40E-01	6.22E-01	6.22E-01	9.82E-01	
WBC count (TLC) (R.R 4-11 × 10^9/l)	6.7	0.3	7.3	0.2	7.53E-01	1.33E-01	1.09E-01	1.09E-01	9.30E-01	
Neutrophils (R.R 40-75%)	52.7	2.5	52.6	2.6	3.07E-02	9.77E-01	9.80E-01	9.77E-01	9.83E-01	
Lymphocytes (R.R 20-50%)	34.4	2.6	35.0	2.7	5.45E-01	8.40E-01	8.88E-01	8.88E-01	9.83E-01	
Monocytes (R.R 2-10%)	7.9	0.4	7.7	0.4	4.32E-03	4.88E-01	6.86E-01	4.88E-01	9.82E-01	
Eosinophils (R.R 1-6%)	4.5	0.8	4.3	0.7	1.60E-01	1.00E+00	8.82E-01	8.82E-01	9.83E-01	
Basophils (R.R < 1%)	0.6	0.1	0.5	0.1	1.19E-01	2.29E-01	2.71E-01	2.71E-01	9.82E-01	
Abs. neutrophils (R.R 2-7 × 10^9/l)	3.55	0.25	3.86	0.23	2.39E-02	1.49E-01	3.75E-01	1.49E-01	9.30E-01	
Abs. lymphocytes (R.R 1-3 × 10^9/l)	2.28	0.18	2.57	0.22	3.24E-01	2.66E-01	3.20E-01	3.20E-01	9.82E-01	
Abs. monocytes (R.R 0 2-1 × 10^9/l)	0.53	0.03	0.56	0.03	1.78E-01	4.35E-01	4.58E-01	4.58E-01	9.82E-01	
Abs. eosinophils (R.R 0.02-0.50 × 10^9/l)	0.31	0.07	0.31	0.06	9.53E-03	6.43E-01	9.85E-01	6.43E-01	9.82E-01	
Abs. basophils (R.R 0.02-0.10 × 10^9/l)	0.04	0.00	0.04	0.00	1.15E-02	4.09E-01	4.41E-01	4.09E-01	9.82E-01	
**Vital signs**										
Blood pressure (systolic) (mmHg)	118.3	2.8	117.9	2.3	2.60E-03	9.05E-01	9.09E-01	9.05E-01	9.83E-01	
Blood pressure (diastolic) (mmHg)	74.5	1.6	73.8	1.6	2.75E-02	6.82E-01	7.41E-01	6.82E-01	9.82E-01	
Respiratory rate (breaths/min)	14.2	0.5	13.8	0.3	1.18E-01	5.14E-01	4.79E-01	4.79E-01	9.82E-01	
Heart rate (bpm)	76.8	1.5	75.6	1.4	9.29E-02	5.99E-01	5.47E-01	5.47E-01	9.82E-01	
Temperature (°C)	37.0	0.0	37.0	0.0	NA	NA	NA	NA	NA	

Data are presented as mean and standard error of the mean (SEM). Both Wilcoxon and t-tests were performed. In the 'Considered p.val' column, either the Wilcoxon or t-test p-value is provided, based on the result of the Shapiro–Wilk test for data distribution (if Shapiro p < 0.05, the Wilcoxon p-value is reported; otherwise, the t-test p-value is selected). The considered p-values were then adjusted using FDR (false discovery rate using the Benjamini–Hochberg method), and the corresponding significance levels are indicated in the 'Sign.' Column, * p < 0.05. Hb: Hemoglobin, RBCs: Red Blood Cells, HCT: Hematocrit, MCV: Mean Corpuscular Volume, MCH: Mean Corpuscular Hemoglobin, MCHC: Mean Corpuscular Hemoglobin Concentration, RDW CV: Red Cell Distribution Width Coefficient of Variation, MPV: Mean Platelet Volume, WBC: White Blood Cells, R.R: Reference Range. Blood sample collection and testing were conducted by Chughtai Lab PK (https://chughtailab.com/).

Table S3 A. Safety assessement of Sucrosomial® vitamin B12 supplementation on partcipant's haematology, liver function, renal function and vital signs in the LRH cohort (n=10).

Sucrosomial® B12 (LRH cohort)	Baseline		Day 7		shapiro	wilcoxon	t. student	considered	fdr	Sign.
Parameter	mean	SEM	mean	SEM	p.val	p.val	p.val	p.val		
**Hematology**										
Hb (R.R 11.5-16 g/dl)	12.8	0.5	12.7	0.5	2.67E-01	6.23E-01	8.36E-01	8.36E-01	1.00E+00	
Total RBCs (R.R 4-6 × 10^12/l)	4.5	0.1	4.5	0.1	3.60E-01	9.70E-01	1.00E+00	1.00E+00	1.00E+00	
HCT (R.R 36-46%)	35.7	1.2	35.7	1.2	8.59E-02	6.74E-01	1.00E+00	1.00E+00	1.00E+00	
MCV (R.R 75-95 fl)	79.6	1.9	79.6	2.0	3.82E-03	9.39E-01	1.00E+00	9.39E-01	1.00E+00	
MCH (R.R 26-32 pg)	28.6	0.8	28.3	0.9	4.90E-03	5.88E-01	7.97E-01	5.88E-01	1.00E+00	
MCHC (R.R 30-35 g/dl)	35.8	0.2	35.5	0.3	1.08E-02	3.73E-01	4.26E-01	3.73E-01	1.00E+00	
RDW CV (R.R 11.5-14.5%)	13.8	0.2	13.8	0.3	4.10E-04	9.68E-01	1.00E+00	9.68E-01	1.00E+00	
Platelet count (R.R 150-400 × 10^9/l)	187.9	17.1	196.9	12.7	4.60E-04	2.47E-01	6.78E-01	2.47E-01	1.00E+00	
MPV (R.R 7-11 fl)	12.7	0.6	12.1	0.5	2.17E-02	4.49E-01	4.18E-01	4.49E-01	1.00E+00	
WBC count (TLC) (R.R 4-11 × 10^9/l)	7.6	0.4	7.6	0.3	4.86E-01	8.50E-01	9.72E-01	9.72E-01	1.00E+00	
Neutrophills (R.R 40-75%)	53.9	1.8	57.4	2.1	6.37E-01	1.61E-01	2.19E-01	2.19E-01	1.00E+00	
Lymphocytes (R.R 20-50%)	35.1	1.9	32.2	1.8	2.33E-01	2.55E-01	2.79E-01	2.79E-01	1.00E+00	
Monocytes (R.R 2-10%)	7.1	0.5	6.0	0.4	1.20E-01	1.23E-01	1.29E-01	1.29E-01	1.00E+00	
Eosinophils (R.R 1-6%)	3.9	1.4	4.4	1.9	9.22E-07	9.69E-01	8.33E-01	9.69E-01	1.00E+00	
Abs. neutrophils (R.R 2-7 × 10^9/l)	4.08	0.23	4.16	0.25	5.05E-01	9.12E-01	8.05E-01	8.05E-01	1.00E+00	
Abs. lymphocytes (R.R 1-3 × 10^9/l)	2.69	0.24	2.43	0.16	6.17E-04	6.50E-01	3.94E-01	6.50E-01	1.00E+00	
Abs. monocytes (R.R 0.2-1 × 10^9/l)	0.55	0.06	0.46	0.04	6.14E-02	2.89E-01	2.24E-01	2.24E-01	1.00E+00	
Abs. eosinophils (R.R 0.02-0.50 × 10^9/l)	0.30	0.12	0.34	0.15	7.80E-07	9.09E-01	8.34E-01	9.09E-01	1.00E+00	
**Liver function tests**										
Serum total bilirubin (R.R 0.1-1.2 mg/dL)	0.8	0.1	1.0	0.2	3.16E-01	6.49E-01	5.23E-01	5.23E-01	1.00E+00	
Serum conjugated bilirubin (R.R < 0.5 mg/dL)	0.3	0.0	0.3	0.1	5.33E-02	9.47E-01	7.16E-01	7.16E-01	1.00E+00	
Serum unconjugated bilirubin (R.R 0.1-1 mg/dL)	0.6	0.1	0.7	0.1	2.02E-01	5.61E-01	4.46E-01	4.46E-01	1.00E+00	
Serum ALT (SGPT) (R.R <34 U/L)	35.9	10.2	34.6	9.2	2.80E-02	1.00E+00	9.27E-01	1.00E+00	1.00E+00	
Serum AST (SGOT) (R.R <31 U/L)	31.7	5.5	30.3	4.2	7.84E-03	9.48E-01	8.40E-01	9.48E-01	1.00E+00	
Serum ALP (R.R 44-107 U/L)	75.7	5.1	74.3	4.4	3.98E-01	1.00E+00	8.48E-01	8.48E-01	1.00E+00	
Serum GGT (R.R <33 U/L)	35.9	10.0	32.1	7.3	3.82E-02	1.00E+00	7.70E-01	1.00E+00	1.00E+00	
Serum total protein (R.R 6-8.5 g/dL)	7.1	0.3	6.8	0.2	1.54E-01	5.22E-01	3.70E-01	3.70E-01	1.00E+00	
Serum albumin (R.R 3.5-5.2 g/dL)	4.0	0.1	4.0	0.1	6.10E-02	6.98E-01	8.34E-01	8.34E-01	1.00E+00	
Serum globulins (R.R 1.8-3.5 g/dL)	3.1	0.3	2.8	0.1	6.06E-02	3.55E-01	3.01E-01	3.01E-01	1.00E+00	
A/G ratio (R.R 1-2.2)	1.3	0.1	1.5	0.1	1.74E-01	4.38E-01	4.93E-01	4.93E-01	1.00E+00	
**Renal function tests**										
Serum urea (R.R 0-50 mg/dl)	25.7	2.3	33.4	3.3	7.91E-01	1.28E-01	8.11E-02	8.11E-02	1.00E+00	
Serum BUN (R.R 8-22 mg/dl)	12.0	1.0	15.6	1.5	7.66E-01	1.24E-01	7.77E-02	7.77E-02	1.00E+00	
Serum creatinine (R.R 0.73- 18 mg/dl)	0.62	0.05	0.64	0.04	3.65E-01	7.49E-01	8.09E-01	8.09E-01	1.00E+00	
eGFR (R.R > 60 ml/min//1.73m2)	121.3	3.0	119.1	2.8	7.55E-02	6.08E-01	6.11E-01	6.11E-01	1.00E+00	
**Vital signs**										
Blood pressure (systolic) (mm Hg)	111.0	2.3	111.7	3.3	2.41E-01	1.00E+00	8.64E-01	8.64E-01	1.00E+00	
Blood pressure (diastolic) (mm Hg)	80.0	2.1	78.9	1.4	1.12E-02	5.99E-01	6.69E-01	5.99E-01	1.00E+00	
Respiratory rate (breaths/min)	15.9	0.7	15.8	0.8	1.51E-01	8.17E-01	9.26E-01	9.26E-01	1.00E+00	
Heart rate (bpm)	87.3	2.7	85.4	2.6	6.52E-02	5.08E-01	6.19E-01	6.19E-01	1.00E+00	
Temperature (°C)	37.0	0.0	37.0	0.0	NA	NA	NA	NA	NA	

Data are presented as mean and standard error of the mean (SEM). Both Wilcoxon and t-tests were performed. In the 'Considered p.val' column, either the Wilcoxon or t-test p-value is provided, based on the result of the Shapiro–Wilk test for data distribution (if Shapiro $p < 0.05$, the Wilcoxon p-value is reported; otherwise, the t-test p-value is selected). The considered p-values were then adjusted using FDR (false discovery rate using the Benjamini–Hochberg method), and the corresponding significance levels are indicated in the 'Sign.' column. Hb: Hemoglobin, RBCs: Red Blood Cells, HCT: Hematocrit, MCV: Mean Corpuscular Volume, MCH: Mean Corpuscular Hemoglobin, MCHC: Mean Corpuscular Hemoglobin Concentration, RDW CV: Red Cell Distribution Width Coefficient of Variation, MPV: Mean Platelet Volume, WBC: White Blood Cells, ALT: Alanine Aminotransferase, AST: Aspartate Aminotransferase, ALP: Alkaline Phosphatase, GGT: Gamma-Glutamyl Transferase, A/G ratio: Albumin/Globulin ratio, BUN: Blood Urea Nitrogen, eGFR: Estimated Glomerular Filtration Rate, R.R: Reference Range. Blood sample collection and testing were conducted by Chughtai Lab PK (https://chughtailab.com/).

Table S3 B. Safety assessement of conventional B-SUB B12 supplementation on partcipant's haematology, liver function, renal function and vital signs in the LRH cohort (n=12).

B-SUB B12 (LRH cohort) Parameter	Baseline mean	Baseline SEM	Day 7 mean	Day 7 SEM	shapiro p.val	wilcoxon p.val	t. student p.val	considered p.val	fdr	Sign.
**Haematology**										
Hb (R.R 11.5-16 g/dl)	13.0	0.5	13.1	0.5	7.60E-01	8.78E-01	9.30E-01	9.30E-01	1.00E+00	
Total RBCs (R.R 4-6 × 10^12/l)	4.8	0.2	4.9	0.2	6.50E-03	5.16E-01	7.55E-01	5.16E-01	1.00E+00	
HCT (R.R 36-46%)	36.7	1.4	37.2	1.2	6.23E-01	7.34E-01	7.80E-01	7.80E-01	1.00E+00	
MCV (R.R 75-95 fl)	77.3	3.1	76.7	3.5	7.24E-04	9.51E-01	9.12E-01	9.51E-01	1.00E+00	
MCH (R.R 26-32 pg)	27.4	1.2	27.0	1.3	6.77E-03	7.56E-01	8.15E-01	7.56E-01	1.00E+00	
MCHC (R.R 30-35 g/dl)	34.0	1.6	35.3	0.1	1.02E-08	6.49E-01	4.38E-01	6.49E-01	1.00E+00	
RDW CV (R.R 11.5-14.5%)	15.8	1.8	14.2	0.4	5.77E-08	1.00E+00	4.12E-01	1.00E+00	1.00E+00	
Platelet count (R.R 150-400 × 10^9/l)	239.4	17.1	261.3	23.1	3.81E-01	5.25E-01	4.57E-01	4.57E-01	1.00E+00	
MPV (R.R 7-11 fl)	10.8	0.4	10.9	0.6	1.21E-02	1.00E+00	9.18E-01	1.00E+00	1.00E+00	
WBC count (TLC) (R.R 4-11 × 10^9/l)	8.2	0.4	9.3	0.6	9.71E-01	1.76E-01	1.92E-01	1.92E-01	1.00E+00	
Neutrophills (R.R 40-75%)	56.7	2.3	57.0	2.7	4.41E-01	7.11E-01	9.26E-01	9.26E-01	1.00E+00	
Lymphocytes (R.R 20-50%)	33.8	2.3	32.5	2.1	7.01E-01	9.02E-01	6.83E-01	6.83E-01	1.00E+00	
Monocytes (R.R 2-10%)	6.1	0.4	6.7	0.6	9.26E-02	5.03E-01	3.82E-01	3.82E-01	1.00E+00	
Eosinophils (R.R 1-6%)	3.5	0.8	3.8	0.9	3.94E-04	8.50E-01	7.96E-01	8.50E-01	1.00E+00	
Abs. neutrophils (R.R 2-7 × 10^9/l)	4.73	0.39	5.40	0.57	4.63E-01	4.24E-01	3.42E-01	3.42E-01	1.00E+00	
Abs. lymphocytes (R.R 1-3 × 10^9/l)	2.68	0.11	2.89	0.20	3.30E-01	3.89E-01	3.79E-01	3.79E-01	1.00E+00	
Abs. monocytes (R.R 0.2-1 × 10^9/l)	0.51	0.06	0.61	0.05	6.83E-01	1.57E-01	2.15E-01	2.15E-01	1.00E+00	
Abs. eosinophils (R.R 0.02-0.50 × 10^9/l)	0.30	0.07	0.35	0.08	1.88E-03	6.22E-01	6.84E-01	6.22E-01	1.00E+00	
**Liver function tests**										
Serum total bilirubin (R.R 0.1-1.2 mg/dL)	0.7	0.1	0.8	0.1	1.98E-02	8.53E-01	9.05E-01	8.53E-01	1.00E+00	
Serum conjugated bilirubin (R.R < 0.5 mg/dL)	0.2	0.0	0.2	0.0	1.44E-03	8.21E-01	9.43E-01	8.21E-01	1.00E+00	
Serum unconjugated bilirubin (R.R 0.1-1 mg/dL)	0.5	0.1	0.5	0.1	3.80E-02	8.52E-01	8.93E-01	8.52E-01	1.00E+00	
Serum ALT (SGPT) (R.R <34 U/L)	29.3	4.4	27.0	4.3	2.00E-03	8.05E-01	7.19E-01	8.05E-01	1.00E+00	
Serum AST (SGOT) (R.R <31 U/L)	27.8	2.2	25.2	2.2	3.07E-02	3.54E-01	4.01E-01	3.54E-01	1.00E+00	
Serum ALP (R.R 44-107 U/L)	80.8	4.6	84.5	5.3	6.11E-02	6.00E-01	6.02E-01	6.02E-01	1.00E+00	
Serum GGT (R.R <33 U/L)	25.0	3.9	20.8	3.5	1.29E-05	1.02E-01	4.33E-01	1.02E-01	1.00E+00	
Serum total protein (R.R 6-8.5 g/dL)	7.2	0.1	7.2	0.1	3.69E-02	8.52E-01	8.99E-01	8.52E-01	1.00E+00	
Serum albumin (R.R 3.5-5.2 g/dL)	4.1	0.1	4.5	0.3	1.15E-06	3.37E-01	2.54E-01	3.37E-01	1.00E+00	
Serum globulins (R.R 1.8-3.5 g/dL)	3.0	0.1	3.0	0.1	6.24E-01	7.80E-01	7.03E-01	7.03E-01	1.00E+00	
A/G ratio (R.R 1-2.2)	1.4	0.1	1.4	0.1	1.64E-01	7.54E-01	7.93E-01	7.93E-01	1.00E+00	
**Renal function tests**										
Serum urea (R.R 0-50 mg/dl)	26.1	2.3	25.5	2.6	3.99E-03	4.77E-01	8.58E-01	4.77E-01	1.00E+00	
Serum BUN (R.R 8-22 mg/dl)	12.3	1.1	11.7	1.2	7.56E-03	4.56E-01	7.50E-01	4.56E-01	1.00E+00	
Serum creatinine (R.R 0.73- 18 mg/dl)	0.64	0.04	0.66	0.04	5.18E-01	5.37E-01	6.71E-01	6.71E-01	1.00E+00	
eGFR (R.R > 60 ml/min//1.73m2)	124.0	2.3	112.6	11.3	4.62E-08	4.59E-01	3.48E-01	4.59E-01	1.00E+00	
**Vital signs**										
Blood pressure (systolic) (mm Hg)	109.2	2.3	110.6	2.5	1.81E-02	5.39E-01	6.78E-01	5.39E-01	1.00E+00	
Blood pressure (diastolic) (mm Hg)	71.7	2.1	74.6	1.7	5.29E-02	2.77E-01	2.89E-01	2.89E-01	1.00E+00	
Respiratory rate (breaths/min)	13.5	0.7	14.2	0.9	6.27E-05	6.96E-01	5.69E-01	6.96E-01	1.00E+00	
Heart rate (bpm)	86.8	3.3	86.0	3.3	5.46E-03	9.53E-01	8.72E-01	9.53E-01	1.00E+00	
Temperature (°C)	37.0	0.0	37.0	0.0	NA	NA	NA	NA	NA	

Data are presented as mean and standard error of the mean (SEM). Both Wilcoxon and t-tests were performed. In the 'Considered p.val' column, either the Wilcoxon or t-test p-value is provided, based on the result of the Shapiro–Wilk test for data distribution (if Shapiro $p < 0.05$, the Wilcoxon p-value is reported; otherwise, the t-test p-value is selected). The considered p-values were then adjusted using FDR (false discovery rate using the Benjamini–Hochberg method), and the corresponding significance levels are indicated in the 'Sign.' column. Hb: Hemoglobin, RBCs: Red Blood Cells, HCT: Hematocrit, MCV: Mean Corpuscular Volume, MCH: Mean Corpuscular Hemoglobin, MCHC: Mean Corpuscular Hemoglobin Concentration, RDW CV: Red Cell Distribution Width Coefficient of Variation, MPV: Mean Platelet Volume, WBC: White Blood Cells, ALT: Alanine Aminotransferase, AST: Aspartate Aminotransferase, ALP: Alkaline Phosphatase, GGT: Gamma-Glutamyl Transferase, A/G ratio: Albumin/Globulin ratio, BUN: Blood Urea Nitrogen, eGFR: Estimated Glomerular Filtration Rate, R.R: Reference Range. Blood sample collection and testing were conducted by Chughtai Lab PK (https://chughtailab.com/).

Table S4 A. Safety assessement of Sucrosomial® vitamin B12 supplementation on partcipant's haematology, liver function, renal function and vital signs in the LUMHS cohort (n=10).

Sucrosomial® B12 (LUMHS cohort)	Baseline		Day 7		shapiro	wilcoxon	t. student	considere	fdr	Sign.
**Parameter**	**mean**	**SEM**	**mean**	**SEM**	**p.val**	**p.val**	**p.val**	**d p.val**		
**Haematology**										
Hb (R.R 14-18 gm/dl)	12.2	0.4	13.5	0.5	1.52E-01	4.89E-02	7.97E-02	7.97E-02	8.01E-01	
HCT (R.R 42-52%)	38.4	1.3	39.4	1.0	2.69E-01	5.29E-01	5.47E-01	5.47E-01	9.38E-01	
RBCs (R.R 4.3-5.9 × 10^6/dl)	4.8	0.2	4.8	0.2	7.27E-01	6.84E-01	8.48E-01	8.48E-01	9.38E-01	
MCV (R.R 76-96 FL)	81.3	3.8	82.3	3.8	1.83E-01	8.20E-01	8.57E-01	8.57E-01	9.38E-01	
MCH (R.R 27-32 PG)	25.6	1.4	28.3	1.4	2.58E-01	1.65E-01	1.81E-01	1.81E-01	8.01E-01	
MCHC (R.R 32-36 g/dL)	31.4	0.7	34.2	0.8	5.85E-01	1.39E-02	1.91E-02	1.91E-02	5.92E-01	
WBC (R.R 4-10 × 10^3/UL)	7.1	0.7	8.4	1.1	2.83E-02	5.79E-01	3.35E-01	5.79E-01	9.38E-01	
Neutrophils (R.R 40-75%)	57.6	1.7	59.7	3.9	9.06E-03	8.48E-01	6.42E-01	8.48E-01	9.38E-01	
Lymphocytes (R.R 20-45%)	35.3	1.6	32.4	3.5	6.26E-03	8.78E-01	4.73E-01	8.78E-01	9.38E-01	
Monocytes (R.R 2-10%)	4.3	0.7	6.1	1.0	2.95E-04	1.72E-01	1.52E-01	1.72E-01	8.01E-01	
Eosinophils (R.R 1-6%)	2.7	0.4	2.0	0.3	3.04E-04	1.59E-01	1.39E-01	1.59E-01	8.01E-01	
Basophils (R.R <1%)	0.1	0.1	0.3	0.1	1.02E-04	1.24E-01	1.68E-01	1.24E-01	8.01E-01	
Platelet count (R.R 150-400 × 10^9/L)	217.2	19.1	239.1	17.9	9.44E-02	3.53E-01	4.13E-01	4.13E-01	9.38E-01	
ESR (R.R 0-25 mm/1Hr)	23.5	5.6	20.9	4.3	1.42E-02	9.09E-01	7.17E-01	9.09E-01	9.39E-01	
**Liver function tests**										
Serum billirubin (total) (R.R 0.10-1 mg/dl)	0.56	0.13	0.56	0.09	1.35E-02	6.84E-01	9.63E-01	6.84E-01	9.38E-01	
Serum billirubin (direct) (R.R <= 0.30 mg/dl)	0.22	0.05	0.16	0.02	3.67E-04	7.39E-01	3.39E-01	7.39E-01	9.38E-01	
Serum billirubin (indirect) (R.R 0.25-0.9 mg/dl)	0.34	0.07	0.39	0.07	2.12E-02	4.72E-01	6.20E-01	4.72E-01	9.38E-01	
SGPT (ALT) (R.R < 45 U/L)	41.1	7.8	43.4	7.4	1.61E-02	8.20E-01	8.33E-01	8.20E-01	9.38E-01	
ALP (R.R 42-129 U/L)	98.9	5.7	103.7	5.3	8.36E-01	5.70E-01	5.45E-01	5.45E-01	9.38E-01	
GGT (R.R 8-61 U/L)	30.6	6.1	34.3	5.8	1.95E-01	6.50E-01	6.64E-01	6.64E-01	9.38E-01	
**Renal function tests**										
Serum creatinine (R.R 0.70-1.20 mg/dl)	0.80	0.05	0.78	0.04	1.91E-01	7.05E-01	7.46E-01	7.46E-01	9.38E-01	
Serum sodium (R.R 136-145 mEq/L)	132.7	3.1	136.9	0.6	2.66E-07	1.17E-01	2.20E-01	1.17E-01	8.01E-01	
Serum potassium (R.R 3.5-5.10 mEq/L)	4.5	0.2	4.4	0.1	3.89E-03	7.31E-01	6.41E-01	7.31E-01	9.38E-01	
Serum chloride (R.R 98-107 mEq/L)	98.9	0.8	99.5	0.4	5.02E-02	1.74E-01	5.29E-01	5.29E-01	9.38E-01	
Serum bicarbonate (R.R 25-29 mEq/L)	26.2	0.4	26.2	0.4	9.02E-02	8.44E-01	1.00E+00	1.00E+00	1.00E+00	
Blood urea (R.R 15-50 mg/dl)	21.3	2.3	21.7	2.2	4.39E-02	8.50E-01	9.02E-01	8.50E-01	9.38E-01	
**Vital signs**										
Blood pressure (systolic) (mm Hg)	125.1	5.2	123.1	4.0	6.82E-01	6.75E-01	7.64E-01	7.64E-01	9.38E-01	
Blood pressure (diastolic) (mm Hg)	80.9	2.9	79.8	2.6	1.73E-01	4.95E-01	7.81E-01	7.81E-01	9.38E-01	
Respiratory rate (breaths/min)	15.4	0.9	15.1	0.9	3.21E-02	7.59E-01	8.17E-01	7.59E-01	9.38E-01	
Heart rate (bpm)	85.9	3.5	84.1	2.6	3.45E-02	5.20E-01	6.82E-01	5.20E-01	9.38E-01	
Temperature (°C)	37.0	0.0	37.0	0.0	NA	NA	NA	NA	NA	

Data are presented as mean and standard error of the mean (SEM). Both Wilcoxon and t-tests were performed. In the 'Considered p.val' column, either the Wilcoxon or t-test p-value is provided, based on the result of the Shapiro–Wilk test for data distribution (if Shapiro $p < 0.05$, the Wilcoxon p-value is reported; otherwise, the t-test p-value is selected). The considered p-values were then adjusted using FDR (false discovery rate using the Benjamini–Hochberg method), and the corresponding significance levels are indicated in the 'Sign.' column. Hb: Hemoglobin, RBCs: Red Blood Cells, HCT: Hematocrit, MCV: Mean Corpuscular Volume, MCH: Mean Corpuscular Hemoglobin, MCHC: Mean Corpuscular Hemoglobin Concentration, WBC: White Blood Cells, ESR: Erythrocyte Sedimentation Rate, SGPT: Serum Glutamic Pyruvic Transaminase, ALT: Alanine Aminotransferase, AST: Aspartate Aminotransferase, ALP: Alkaline Phosphatase, GGT: Gamma-Glutamyl Transferase, R.R: Reference Range. Blood sample collection and testing were conducted by Diagnostic and Research Laboratory, LUMHS, PK (https://drlab.lumhs.edu.pk/).

Table S4 B. Safety assessement of conventional Evermin B12 supplementation on partcipant's haematology, liver function, renal function and vital signs in the LUMHS cohort (n=10).

Evermin B12 (LUMHS cohort)	Baseline		Day 7		shapiro	wilcoxon	t. student	considered	fdr	Sign.
Parameter	mean	SEM	mean	SEM	p.val	p.val	p.val	p.val		
**Haematology**										
Hb (R.R 14-18 gm/dl)	12.9	0.5	13.8	0.5	2.91E-01	3.64E-01	2.38E-01	2.38E-01	9.55E-01	
HCT (R.R 42-52%)	39.4	1.4	39.7	1.0	1.25E-02	7.61E-01	8.70E-01	7.61E-01	9.55E-01	
RBCs (R.R 4.3-5.9 × 10^6/dl)	4.8	0.1	4.8	0.1	2.85E-01	9.10E-01	8.38E-01	8.38E-01	9.55E-01	
MCV (R.R 76-96 FL)	82.5	2.6	83.1	2.6	3.67E-04	7.62E-01	8.65E-01	7.62E-01	9.55E-01	
MCH (R.R 27-32 PG)	27.1	1.1	28.9	1.1	6.21E-02	1.73E-01	2.71E-01	2.71E-01	9.55E-01	
MCHC (R.R 32-36 g/dL)	32.7	0.5	34.7	0.6	6.36E-01	4.91E-02	1.59E-02	1.59E-02	2.46E-01	
WBC (R.R 4-10 × 10^3/UL)	8.0	0.7	7.8	0.6	4.18E-01	9.10E-01	8.75E-01	8.75E-01	9.55E-01	
Neutrophils (R.R 40-75%)	58.6	3.2	57.8	3.9	5.72E-01	9.39E-01	8.76E-01	8.76E-01	9.55E-01	
Lymphocytes (R.R 20-45%)	30.8	2.6	32.2	3.3	8.34E-01	7.05E-01	7.43E-01	7.43E-01	9.55E-01	
Monocytes (R.R 2-10%)	5.7	0.8	5.2	0.7	2.97E-02	8.78E-01	6.71E-01	8.78E-01	9.55E-01	
Eosinophils (R.R 1-6%)	4.7	1.0	4.7	1.1	3.72E-04	7.25E-01	9.79E-01	7.25E-01	9.55E-01	
Basophils (R.R <1%)	0.3	0.1	0.2	0.1	2.87E-05	6.57E-01	5.67E-01	6.57E-01	9.55E-01	
Platelet count (R.R 150-400 × 10^9/L)	226.3	16.2	222.8	11.9	3.03E-01	6.50E-01	8.64E-01	8.64E-01	9.55E-01	
ESR (R.R 0-25 mm/1Hr)	27.0	5.4	17.5	3.7	4.01E-03	1.24E-01	1.64E-01	1.24E-01	9.55E-01	
**Liver function tests**										
Serum bilirubin (total) (R.R 0.10-1 mg/dl)	0.43	0.06	0.44	0.06	9.61E-01	8.50E-01	9.35E-01	9.35E-01	9.55E-01	
Serum bilirubin (direct) (R.R <= 0.30 mg/dl)	0.13	0.02	0.15	0.02	4.53E-01	4.81E-01	4.27E-01	4.27E-01	9.55E-01	
Serum bilirubin (indirect) (R.R 0.25-0.9 mg/dl)	0.31	0.05	0.29	0.04	8.19E-01	8.21E-01	8.03E-01	8.03E-01	9.55E-01	
SGPT (ALT) (R.R < 45 U/L)	41.7	8.3	43.0	8.8	1.34E-03	9.40E-01	9.15E-01	9.40E-01	9.55E-01	
ALP (R.R 42-129 U/L)	94.9	9.1	93.9	10.5	4.70E-02	9.40E-01	9.43E-01	9.40E-01	9.55E-01	
GGT (R.R 8-61 U/L)	25.8	8.2	34.9	8.4	1.14E-03	3.43E-01	4.50E-01	3.43E-01	9.55E-01	
**Renal function tests**										
Serum creatinine (R.R 0.70-1.20 mg/dl)	0.83	0.06	0.84	0.05	3.96E-01	9.40E-01	8.76E-01	8.76E-01	9.55E-01	
Serum sodium (R.R 136-145 mEq/L)	136.3	0.7	138.1	1.0	3.17E-01	1.31E-01	1.67E-01	1.67E-01	9.55E-01	
Serum potassium (R.R 3.5-5.10 mEq/L)	4.3	0.1	4.3	0.1	3.26E-01	1.00E+00	9.55E-01	9.55E-01	9.55E-01	
Serum chloride (R.R 98-107 mEq/L)	99.4	0.6	100.6	1.3	1.15E-01	7.02E-01	4.10E-01	4.10E-01	9.55E-01	
Serum bicarbonate (R.R 25-29 mEq/L)	26.2	1.3	25.1	0.7	1.66E-03	1.14E-01	4.82E-01	1.14E-01	9.55E-01	
Blood urea (R.R 15-50 mg/dl)	22.8	2.1	24.1	2.4	8.67E-01	6.49E-01	6.86E-01	6.86E-01	9.55E-01	
**Vital signs**										
Blood pressure (systolic) (mm Hg)	125.8	4.1	127.2	3.6	4.36E-01	9.39E-01	8.02E-01	8.02E-01	9.55E-01	
Blood pressure (diastolic) (mm Hg)	86.4	4.2	85.0	2.5	2.56E-01	7.05E-01	7.78E-01	7.78E-01	9.55E-01	
Respiratory rate (breaths/min)	13.9	0.4	14.6	0.9	2.73E-01	6.19E-01	4.90E-01	4.90E-01	9.55E-01	
Heart rate (bpm)	78.1	2.1	79.5	1.2	7.06E-01	5.95E-01	5.67E-01	5.67E-01	9.55E-01	
Temperature (°C)	37.0	0.0	37.0	0.0	NA	NA	NA	NA	NA	

Data are presented as mean and standard error of the mean (SEM). Both Wilcoxon and t-tests were performed. In the 'Considered p.val' column, either the Wilcoxon or t-test p-value is provided, based on the result of the Shapiro–Wilk test for data distribution (if Shapiro $p < 0.05$, the Wilcoxon p-value is reported; otherwise, the t-test p-value is selected). The considered p-values were then adjusted using FDR (false discovery rate using the Benjamini–Hochberg method), and the corresponding significance levels are indicated in the 'Sign.' column. Hb: Hemoglobin, RBCs: Red Blood Cells, HCT: Hematocrit, MCV: Mean Corpuscular Volume, MCH: Mean Corpuscular Hemoglobin, MCHC: Mean Corpuscular Hemoglobin Concentration, WBC: White Blood Cells, ESR: Erythrocyte Sedimentation Rate, SGPT: Serum Glutamic Pyruvic Transaminase, ALT: Alanine Aminotransferase, AST: Aspartate Aminotransferase, ALP: Alkaline Phosphatase, GGT: Gamma-Glutamyl Transferase, R.R: Reference Range. Blood sample collection and testing were conducted by Diagnostic and Research Laboratory, LUMHS, PK (https://drlab.lumhs.edu.pk/).

Table S4 C. Safety assessement of conventional Neuromax B12 supplementation on partcipant's haematology, liver function, renal function and vital signs in the LUMHS cohort (n=10).

Neuromax B12 (LUMHS cohor)	Baseline		Day 7		shapiro	wilcoxon	t. student	considered	fdr	Sign.
Parameter	mean	SEM	mean	SEM	p.val	p.val	p.val	p.val		
**Haematology**										
Hb (R.R 14-18 gm/dl)	13.3	0.4	13.1	0.5	1.24E-01	6.50E-01	8.00E-01	8.00E-01	8.84E-01	
HCT (R.R 42-52%)	39.8	1.0	38.5	1.2	5.50E-01	4.27E-01	4.36E-01	4.36E-01	8.84E-01	
RBC (R.R 4.3-5.9 × 10^6/dl)	4.7	0.1	4.6	0.2	5.80E-01	6.50E-01	5.47E-01	5.47E-01	8.84E-01	
MCV (R.R 76-96 FL)	84.4	1.9	84.0	2.0	6.00E-01	8.53E-01	8.84E-01	8.84E-01	8.84E-01	
MCH (R.R 27-32 PG)	28.2	0.8	28.6	0.8	5.15E-01	5.29E-01	7.01E-01	7.01E-01	8.84E-01	
MCHC (R.R 32-36 g/dL)	33.3	0.3	34.0	0.3	7.05E-01	1.11E-01	1.08E-01	1.08E-01	4.17E-01	
WBC (R.R 4-10 × 10^3/UL)	8.8	0.7	8.8	0.6	3.61E-02	8.50E-01	9.76E-01	8.50E-01	8.84E-01	
Neutrophils (R.R 40-75%)	60.5	1.8	60.0	2.0	4.16E-01	7.90E-01	8.66E-01	8.66E-01	8.84E-01	
Lymphocytes (R.R 20-45%)	29.5	1.9	33.5	2.2	2.14E-01	3.41E-01	1.92E-01	1.92E-01	6.63E-01	
Monocytes (R.R 2-10%)	7.0	0.5	3.5	0.3	3.74E-03	3.78E-04	2.78E-05	3.78E-04	2.34E-03	**
Eosinophils (R.R 1-6%)	2.6	0.4	3.0	0.8	1.78E-05	6.12E-01	6.75E-01	6.12E-01	8.84E-01	
Basophils (R.R <1%)	0.4	0.1	0.0	0.0	2.85E-04	2.15E-04	2.02E-04	2.15E-04	2.34E-03	**
Platelet count (R.R 150-400 × 10^9/L)	245.2	26.5	231.7	28.3	1.10E-01	4.27E-01	7.32E-01	7.32E-01	8.84E-01	
ESR (R.R 0-25 mm/1Hr)	16.7	2.6	34.0	5.7	1.10E-02	3.49E-02	1.70E-02	3.49E-02	1.54E-01	
**Liver function tests**										
Serum bilirubin (total) (R.R 0.10-1 mg/dl)	0.67	0.17	0.75	0.18	4.62E-05	4.27E-01	7.41E-01	4.27E-01	8.84E-01	
Serum bilirubin (direct) (R.R <= 0.30 mg/dl)	0.23	0.07	0.28	0.05	6.68E-04	2.47E-01	5.68E-01	2.47E-01	7.67E-01	
Serum bilirubin (indirect) (R.R 0.25-0.9 mg/dl)	0.43	0.11	0.47	0.12	5.06E-05	5.96E-01	8.42E-01	5.96E-01	8.84E-01	
SGPT (ALT) (R.R < 45 U/L)	36.9	5.6	35.6	4.5	9.67E-02	9.40E-01	8.59E-01	8.59E-01	8.84E-01	
ALP (R.R 42-129 U/L)	118.6	8.0	114.5	9.1	9.27E-01	9.10E-01	7.39E-01	7.39E-01	8.84E-01	
GGT (R.R 8-61 U/L)	24.5	2.9	22.6	3.2	2.80E-01	7.04E-01	6.66E-01	6.66E-01	8.84E-01	
**Kidney function tests**										
Serum creatinine (R.R 0.70-1.20 mg/dl)	0.88	0.03	0.91	0.03	1.26E-01	5.20E-01	5.17E-01	5.17E-01	8.84E-01	
Serum sodium (R.R 136-145 mEq/L)	136.1	0.4	139.2	0.6	5.23E-01	1.01E-03	2.87E-04	2.87E-04	2.34E-03	**
Serum potassium (R.R 3.5-5.10 mEq/L)	4.2	0.1	4.9	0.1	4.38E-01	8.19E-04	3.42E-04	3.42E-04	2.34E-03	**
Serum chloride (R.R 98-107 mEq/L)	98.5	0.3	102.3	0.8	1.01E-01	1.33E-03	5.97E-04	5.97E-04	3.09E-03	**
Serum bicarbonate (R.R 25-29 mEq/L)	28.0	0.3	20.8	0.7	2.16E-02	1.59E-04	4.03E-07	1.59E-04	2.34E-03	**
Blood urea (R.R 15-50 mg/dl)	22.4	2.2	23.1	2.5	2.13E-02	6.49E-01	8.34E-01	6.49E-01	8.84E-01	
**Vital signs**										
Blood pressure (systolic) (mm Hg)	120.0	4.7	118.5	4.5	4.44E-02	8.48E-01	8.20E-01	8.48E-01	8.84E-01	
Blood pressure (diastolic) (mm Hg)	77.0	2.1	75.7	2.1	1.03E-03	7.16E-01	6.70E-01	7.16E-01	8.84E-01	
Respiratory rate (breaths/min)	18.0	1.3	19.4	0.4	1.86E-06	5.61E-01	3.26E-01	5.61E-01	8.84E-01	
Heart rate (bpm)	81.2	2.3	83.7	2.2	1.18E-01	4.46E-01	4.43E-01	4.43E-01	8.84E-01	
Temperature (°C)	37.0	0.0	37.0	0.0	NA	NA	NA	NA	NA	

Data are presented as mean and standard error of the mean (SEM). Both Wilcoxon and t-tests were performed. In the 'Considered p.val' column, either the Wilcoxon or t-test p-value is provided, based on the result of the Shapiro–Wilk test for data distribution (if Shapiro $p < 0.05$, the Wilcoxon p-value is reported; otherwise, the t-test p-value is selected). The considered p-values were then adjusted using FDR (false discovery rate using the Benjamini–Hochberg method), and the corresponding significance levels are indicated in the 'Sign.' column. Hb: Hemoglobin, RBCs: Red Blood Cells, HCT: Haematocrit, MCV: Mean Corpuscular Volume, MCH: Mean Corpuscular Haemoglobin, MCHC: Mean Corpuscular Haemoglobin Concentration, WBC: White Blood Cells, ESR: Erythrocyte Sedimentation Rate, SGPT: Serum Glutamic Pyruvic Transaminase, ALT: Alanine Aminotransferase, AST: Aspartate Aminotransferase, ALP: Alkaline Phosphatase, GGT: Gamma-Glutamyl Transferase, R.R: Reference Range. Blood sample collection and testing were conducted by Diagnostic and Research Laboratory, LUMHS, PK (https://drlab.lumhs.edu.pk/).
